# A nomogram to predict prolonged stay of obesity patients with sepsis in ICU: Relevancy for predictive, personalized, preventive, and participatory healthcare strategies

**DOI:** 10.3389/fpubh.2022.944790

**Published:** 2022-08-11

**Authors:** Yang Chen, Mengdi Luo, Yuan Cheng, Yu Huang, Qing He

**Affiliations:** Department of Intensive Care Medicine, Affiliated Hospital of Southwest Jiaotong University/The Third People's Hospital of Chengdu, Chengdu, China

**Keywords:** nomogram, obesity, sepsis, ICU stay, eICU, MIMIC-IV

## Abstract

**Objective:**

In an era of increasingly expensive intensive care costs, it is essential to evaluate early whether the length of stay (LOS) in the intensive care unit (ICU) of obesity patients with sepsis will be prolonged. On the one hand, it can reduce costs; on the other hand, it can reduce nosocomial infection. Therefore, this study aimed to verify whether ICU prolonged LOS was significantly associated with poor prognosis poor in obesity patients with sepsis and develop a simple prediction model to personalize the risk of ICU prolonged LOS for obesity patients with sepsis.

**Method:**

In total, 14,483 patients from the eICU Collaborative Research Database were randomized to the training set (3,606 patients) and validation set (1,600 patients). The potential predictors of ICU prolonged LOS among various factors were identified using logistic regression analysis. For internal and external validation, a nomogram was developed and performed.

**Results:**

ICU prolonged LOS was defined as the third quartile of ICU LOS or more for all sepsis patients and demonstrated to be significantly associated with the mortality in ICU by logistic regression analysis. When entering the ICU, seven independent risk factors were identified: maximum white blood cell, minimum white blood cell, use of ventilation, Glasgow Coma Scale, minimum albumin, maximum respiratory rate, and minimum red blood cell distribution width. In the internal validation set, the area under the curve was 0.73, while in the external validation set, it was 0.78. The calibration curves showed that this model predicted probability due to actually observed probability. Furthermore, the decision curve analysis and clinical impact curve showed that the nomogram had a high clinical net benefit.

**Conclusion:**

In obesity patients with sepsis, we created a novel nomogram to predict the risk of ICU prolonged LOS. This prediction model is accurate and reliable, and it can assist patients and clinicians in determining prognosis and making clinical decisions.

## Introduction

With modern intensive care medicine headed toward an era where economy and efficiency are valued, it is becoming increasingly necessary to accelerate the turnover of intensive care unit (ICU) beds and implement planned bed management (1). Intensive care medicine will transition from a reactive to a proactive discipline in a few years, becoming predictive, personalized, preventive, and participatory (P4) (2), to effectively prevent the adverse prognosis of various diseases in ICU.

Sepsis in the ICU continues to be one of the leading causes of life-threatening conditions, leading to dysfunction of vital organs due to a dysregulated host response to infection, and remains a major global public health problem (3–6). It is estimated that ~15–19 million people die from sepsis worldwide each year (7). The incidence and cost of sepsis has been steadily increasing in recent years due to several factors, one being the emergence of drug-resistant and more lethal pathogens, the other being the aging of society and the malnutrition, poverty and lack of access to medicines in developing countries. Often sepsis, especially severe sepsis, requires transfer to an ICU for appropriate medical care. In the United States, the number of severe sepsis cases rose by 71% between 2003 and 2007, and the total cost of all severe sepsis patients grew by 57% over the same period (8). In 2008, the treatment cost of sepsis in the United States was ~$14.6 billion (9). In most cases, the treatment of sepsis is ranked as one of the costliest diseases in any hospital (10–12).

Body Mass Index (BMI) is defined as obesity that is calculated as weight (kg)/height^2^ (m^2^). The World Health Organisation uses the definition of overweight and obesity as a disorder of excess or abnormal fat, which will increase health risks. The prevalence of obesity is constantly increasing throughout the world, with ~20% of ICU patients (13). Compared with normal or underweight, overweight or obesity is associated with significantly higher survival rates, called the obesity paradox. This has been observed in a variety of diseases, including coronary artery disease (14), coronavirus disease 2019 (COVID-19) (15), acute respiratory diseases (16), infection diseases (5, 17), or critical illness in general (13, 18–23). Similarly, this phenomenon also exists in sepsis (6, 24–31). In addition, many studies showed that obesity patients with sepsis have longer hospital or ICU length of stay (LOS) than non-obesity groups (19, 24, 31, 32). For instance, a large retrospective cohort study showed that obesity patients with sepsis tended to ICU prolonged LOS (p-LOS) than non-obesity groups (24). In general, p-LOS not only leads to an increase in hospitalization or ICU costs but also implies an increase in the risk of hospital-acquired infections (33). In the United States and Canada, researchers have confirmed that a reduction in ICU service levels can result in hospital cost savings (34). Early identification and correction of potential risk factors for ICU p-LOS in obesity patients with sepsis is therefore critical from a clinical and financial standpoint.

All in all, the purpose of our retrospective analysis was two-fold. First, based on an extensive database, we evaluated the differences of ICU LOS among different BMI groups and verified whether ICU p-LOS was significantly associated with adverse prognosis in obesity patients with sepsis. Second, if ICU p-LOS does be an independent predictor of poor prognosis in obesity patients with sepsis, we will develop a predictive nomogram, a simple graphical representation of the scoring model. By constructing a multiple variable regression model, such as logistic regression, a score is assigned to each value level of each influencing factor according to the degree of influence of each influencing factor on the outcome variable (the magnitude of the regression coefficient), and then the scores are summed to obtain the total score. Finally, the predicted probability of the outcome event for the individual is calculated through a functional transformation relationship between the total score and the probability of the outcome event. The nomogram transforms complex regression equations into simple and visual graphs, making the results of the prediction model more readable and of greater use. This advantage has led to an increased interest and use of nomograms in medical research and clinical practice in recent years (35). The nomogram can be customized to predict the likelihood of ICU p-LOS in obesity sepsis patients. Thus, assisting physicians and nurses in selecting the appropriate treatment plan, increasing ICU bed turnover, and reducing sepsis-related ICU medical costs.

## Methods

### Data source

We initiated an observational study using data from the eICU Collaborative Research Database (eICU-CRD), which contains deidentified health-related data from over 200,000 admissions to ICUs monitored by The Philips eICU Program at 208 hospitals between 2014 and 2015 across the United States (36). To comply with the US Health Insurance Portability and Accountability Act (HIPAA), all tables in this database are deidentified. Furthermore, our external validation set was derived from the Medical Information Mart for Intensive Care IV database (MIMIC-IV), which contains comprehensive and high-quality hospitalization data admitted to the Higher Medical Center in Boston, Massachusetts, from 2008 to 2019 (37). The requirement for individual patient consent is irrelevant because all data is anonymous. The author, Chen, extracted the data in our study after completing a National Institutes of Health web-based training course and the Protection of Human Research Participants Examination (No. 36328122).

### Study population

The Third International Consensus Definition of Sepsis (Sepsis-3) is the currently accepted diagnostic criteria for sepsis (4), but the eICU-CRD was established well before 2016, and microbiological culture results were largely unavailable. Therefore, using the Acute Physiology and Chronic Health Evaluation IV (APACHE IV) diagnostic system in eICU-CRD, we included all patients with a first admission diagnosis of sepsis coded by trained eICU-CRD clinicians (38, 39). The exclusion criteria were as follows: I. missing APACHE IV score; II. missing accurate weight and height records on admission; III. admission < 24 h; IV. sequential organ failure assessment (SOFA) score < 2; V. missing underlying information such as sex. Subsequently, we excluded non-obesity patients (BMI < 30 kg/m^2^) from the first cohort as the second cohort. In addition, to independently assess predictive model performance, the MIMIC-IV obesity sepsis cohort was used as an external validation set, with similar exclusion criteria.

### Data extraction

We used PgAdmin (version 4.24) to run structure query language (SQL) to extract data from eICU-CRD. We retrospectively collected the following data: (1) demographic data: age, gender, ethnicity, admission height and admission weight; (2) sites of infection: pulmonary, renal, gastrointestinal tract, skin/soft tissue; (3) vital signs: heart rates, respiratory rates, mean arterial pressure, temperature; (4) severity score: APACHE IV, SOFA, Glasgow coma scale (GCS); (5) comorbidities: chronic obstructive pulmonary disease, chronic kidney disease, shock history, liver disease, hypertension, congestive heart failure, coronary heart disease, malignant tumors, diabetes, which were encoded and defined in the APACHE IV diagnosis system or in the International Classification of Diseases, Ninth/Ten Revision (ICD-9/10); (6) laboratory results: sodium, potassium, hemoglobin, calcium, red cell volume distribution width (RDW), white blood cell, creatinine, lactate, blood urea nitrogen (BUN), albumin, red blood cell (RBC), platelet, mean red cell volume (MCV), glucose, bilirubin, bicarbonate, chloride; (7) intervention-associated information: ventilation, vasopressors, dialysis. Our inclusion period is 24 h before and after entering the ICU. For vital signs and laboratory results, we kept their maximum and minimum values.

### Outcomes

We extracted the following outcome variables: (1) ICU LOS; (2) hospital LOS; (3) all-cause ICU mortality; (4) all-cause hospital mortality. ICU LOS was the primary endpoint; others were the secondary endpoints. Furthermore, the secondary endpoints were extracted solely for the purpose of descriptive analysis.

### Statistical analysis

Our statistical analyses and graphs were conducted in SPSS for Windows (Version 26.0), MedCalc (Version 20.015), GraphPad (Version 9.0.0) and R (Version 4.0.3). Continuous variables were described as mean ± standard deviation (SD) or median with interquartile range (IQR). Patient's demographic and clinical characteristics were compared using Student' *t*-test, Mann–Whitney U test or chi-squared test, as appropriate.

The first cohort was the cohort obtained by excluding all sepsis admissions from the EICU database by our exclusion criteria. In the first cohort, we compared the ICU LOS of four groups by one-way analysis of variance (ANOVA) to confirm whether sepsis patients in the obesity group had a trend toward longer ICU LOS.

The second cohort was the cohort obtained by including obesity patients of the first cohort. In the second cohort, logistic regression analysis was used to see if ICU p-LOS was an independent risk factor for sepsis-related ICU mortality. In addition, to reduce the interference of data deviation and confounding factors, propensity score matching (PSM) was performed between the ICU p-LOS and non-ICU p-LOS cohorts (40).

Obesity patients with sepsis in the second cohort were randomly assigned to a training set and an internal validation set in a 7:3 proportion during the nomogram development process. Then, in the training set, we used univariate logistic regression analysis to screen for variables linked to ICU p-LOS, and the magnitude of the relationship was measured using an odds ratio (OR) with a 95% confidence interval (CI). APACHE IV and SOFA were excluded from the nomogram because they were collinear with other variables in our study. Furthermore, the infection sites were left out of the model because they were dependent on microbial culture, which was a time-consuming process. After that, a stepwise multivariate logistic regression analysis was used to identify independent risk factors for predicting ICU p-LOS by selecting variables with univariate *P*-values 0.05. We used the variance inflation factor (VIF) to test for collinearity between continuous variables, and arithmetic square root of VIF 2 was considered non-collinearity. The Hosmer-Lemeshow test was used to assess the logistic regression model's fitness, with a *P*-value of >0.05 indicating a good fit. Based on the multivariate logistic regression analysis result, we would build a model to achieve the research purpose with fewer variables according to Occam's razor law (41). Finally, based on our model, using R software with RMS package (version 6.2-0) and DynNom package (version 5.0.1), the conventional nomogram and the newly developed interactive web dynamic nomogram were obtained by the training set. The area under the curve (AUC) of the receiver operating characteristic (ROC) curves and calibration with the bootstrap method with 1,000 resampling were used to evaluate the nomogram's performance in all sets. In addition, to compare discrimination slopes and evaluate model fitness, we calculated the integrated discrimination improvement (IDI). Then, in all sets, we used Decision Curve Analysis (DCA) and generated Clinical Impact Curves (CIC) to assess the net benefit of medical interventions that conformed to the nomogram at various threshold probabilities. All analyses were presented in the form of a transparent multivariable prediction model for individual prognosis or diagnosis (TRIPOD) (42). The maximum missing value of all variables did not exceed 25%. Multiple interpolation processes the missing values in logistic regression and model construction. All reported *P*-values were two-tailed, and statistical significance was defined as *P* < 0.05.

## Results

### Comparison of sepsis patients based on BMI classification

**Figure 1:**
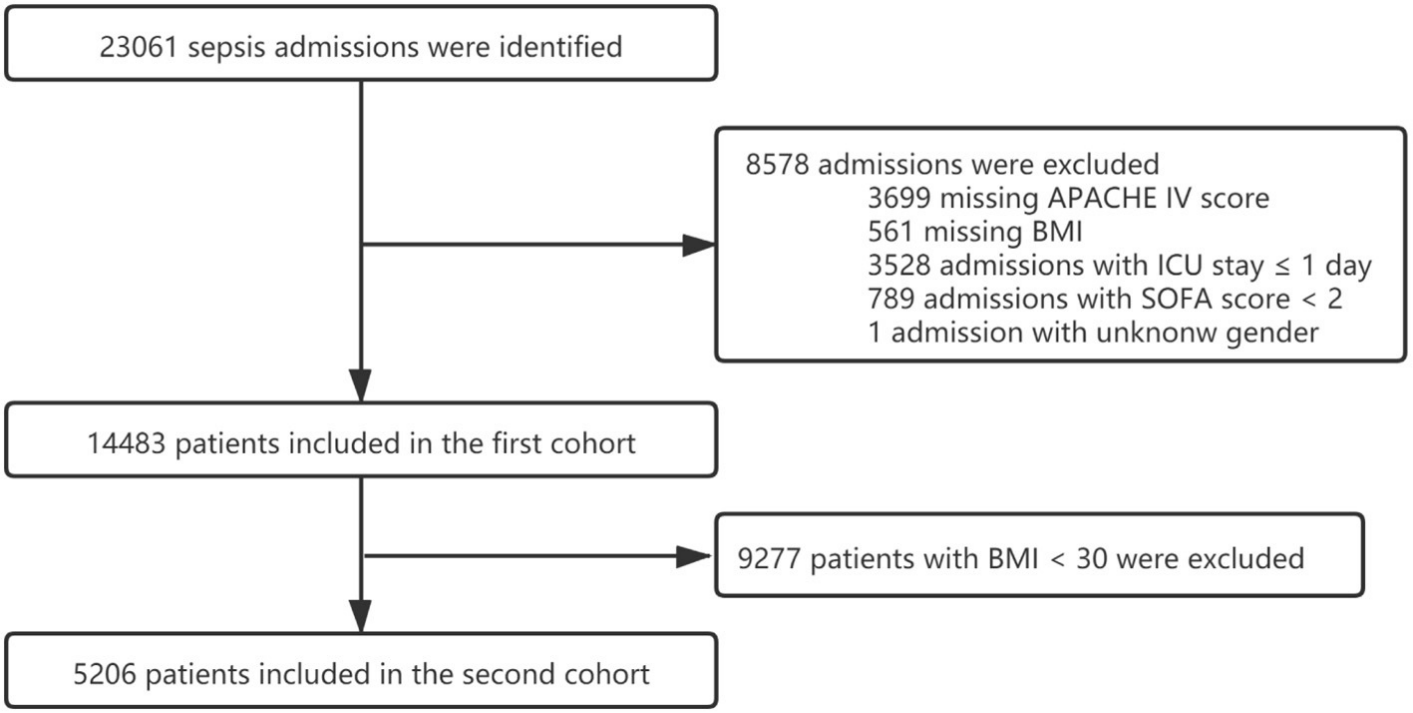
Flow chart of patient screening.

After being screened using the inclusion and exclusion criteria (Figure 1), a total of 14,483 sepsis patients were included in the first cohort (Table 1). We divided all sepsis patients into four groups based on the WHO BMI classification standard for obesity categories: underweight (18.5 kg/m^2^), normal weight (18.5–24.9 kg/m^2^), overweight (25–29.9 kg/m^2^), and obesity (30 kg/m^2^). We found that the median ICU LOS of obesity group was [2.97 days (IQR, 1.87–5.56 days)] which was significantly longer than the other three groups (*P* < 0.001) (Supplementary Table S1). In EICU, the third quartile value of ICU LOS for all sepsis patients was about 5 days; thus, patients with ICU LOS of 5 days or more were considered to have ICU p-LOS in training set and internal validation set. The results showed that the proportion of ICU p-LOS in the obesity group was 28.43% which was also significantly more than the other groups (*p* < 0.001). Furthermore, the results revealed that the obesity group made up the largest proportion of the overall cohort, accounting for more than half of the total. Obesity patients were younger and had the lowest male-to-female ratio. Notably, the obesity group had significantly lower ICU and hospital mortality rates than the non-obesity groups.

**Table 1:** Baseline characteristics of patients on ICU admission (*n* = 14,483).

**Characteristics**	**Total**	**G1**	**G2**	**G3**	**G4**	***P*** **-value**
		***N*** ** = 827**	***N*** ** = 4,626**	***N*** ** = 3,808**	***N*** ** = 5,206**	
Age, years	66.0 (57.0, 78.0)	66.0 (56.0, 81.0)	67.0 (58.0, 81.0)	68.0 (58.0, 79.0)	64.0 (55.0, 74.0)	<0.001
Male, *n* (%)	7,465.0 (51.5)	408.0 (49.3)	2,540.0 (54.7)	2,084.0 (54.7)	2,433.0 (46.7)	<0.001
Race, *n* (%)						<0.001
Caucasian	11,360.0 (78.4)	628.0 (75.9)	3,596.0 (77.5)	2,983.0 (78.3)	4,153.0 (79.8)	
American	1,627.0 (11.2)	123.0 (14.9)	502.0 (10.8)	397.0 (10.4)	605.0 (11.6)	
Other/unknown	1,496.0 (10.3)	76.0 (9.2)	544.0 (11.7)	428.0 (11.2)	448.0 (8.6)	
Height, cm	168.0 (160.0, 177)	169.0 (160.0, 178.0)	169.0 (162.0, 178.0)	170.0 (163.0, 178.0)	168.0 (160.0, 177.0)	<0.001
Weight, kg	77.0 (63.0, 96.0)	47.0 (42.0, 53.0)	64.0 (57.0, 70.0)	79.0 (71.0, 86.0)	104.0 (91.0, 119.0)	<0.001
BMI, kg/m^2^	27.1 (22.9, 33.1)	17.2 (16.1, 17.9)	22.3 (20.7, 23.7)	27.3 (26.0, 28.5)	35.6 (32.4, 41.2)	<0.001
**Severity score^a^**						
APACHE IV	68.0 (53.0, 86.0)	71.0 (55.0, 88.0)	69.0 (54.0, 87.0)	69.0 (54.0, 86.0)	66.0 (51.0, 84.0)	<0.001
SOFA	6.0 (4.0, 9.0)	6.0 (4.0, 9.0)	6.0 (4.0, 9.0)	6.0 (4.0, 9.0)	6.0 (4.0, 9.0)	0.386
ICU LOS, days	2.9 (1.8, 5.1)	2.8 (1.7, 4.5)	2.8 (1.8, 5.0)	2.9 (1.8, 5.1)	3.0 (1.8, 5.5)	<0.001
Hospital LOS, days	7.9 (4.9, 13.3)	7.4 (4.8, 12.9)	7.9 (4.9, 13.1)	7.8 (4.9, 13.0)	8.0 (5.0, 13.9)	<0.001
ICU mortality, *n* (%)	1,419.0 (9.8)	112.0 (13.5)	477.0 (10.3)	374.0 (9.8)	456.0 (8.8)	<0.001
Hospital mortality, *n* (%)	2,320.0 (16.0)	186.0 (22.5)	804.0 (17.3)	604.0 (15.9)	726.0 (13.9)	<0.001
ICU p-LOS, *n* (%)	3,828.0 (26.4)	827.0 (22.6)	1,165.0 (25.1)	996.0 (26.2)	1,480.0 (28.4)	<0.001

Data are expressed as median (IQR), or n (%). Analysis of variance (or the Kruskal-Wallis test) and Chi-square (or Fisher's exact) tests were used for comparisons among groups. Statistical significance (P < 0.05); G1, BMI < 18.5; G2, 18.5 > BMI ≤ 25.0; G3, 25.0 > BMI ≤ 30.0; G4, BMI > 30.0.

a Severe score is calculated on the first day of each ICU patients' stay.

BMI, body mass index; APACHE IV, acute physiology and chronic health evaluation IV; SOFA, sequential organ failure assessment; ICU, intensive care unit; LOS, length of stay; p-LOS, prolonged length of stay.

### Characteristics of obesity patients with sepsis

Table 1 showed the characteristics of all obesity participants at baseline and on the first day of ICU admission, as well as participants in the ICU p-LOS and non-ICU p-LOS groups. ICU LOS was prolonged in ~28% of patients, with no significant difference between the two groups in terms of gender, with males accounting for a lower proportion than females. And that patients with ICU p-LOS were more likely to suffer from chronic obstructive pulmonary disease (COPD), chronic kidney disease (CKD), liver disease, hypertension and congestive heart failure (CHF). We also found that ICU mortality of ICU p-LOS and non-ICU p-LOS groups was 188 (12.70%) and 268 (7.19%), respectively, with a statistical difference of *p* < 0.001, while hospital mortality was similar. Furthermore, clinical variables such as the majority of vital signs, severity scores, laboratory results, and intervention use were significantly different between the two groups, indicating significant heterogeneity (Table 2).

**Table 2:** Baseline clinical and laboratory characteristics of obesity patients with sepsis (*n* = 5,206).

**Characteristics**	**Total**	**Non-ICU p-LOS**,	**ICU p-LOS**,	***P***
		***n*** ** = 3,726**	***n*** ** = 1,480**	
Age, years	65.0 (55.0, 74.0)	65.0 (55.0, 75.0)	64.0 (54.0, 73.0)	0.005
Male, n (%)	2,433.0 (46.8)	1,732.0 (46.5)	701.0 (47.4)	0.566
Race, n (%)				0.001
Caucasian	4,153.0 (79.8)	3,019.0 (81.0)	1,134.0 (76.6)	
American	605.0 (11.6)	416.0 (11.2)	189.0 (12.8)	
Other/unknown	448.0 (8.6)	291.0 (7.8)	157.0 (10.6)	
Comorbidities, *n* (%)				
Chronic obstructive pulmonary disease	462.0 (8.8)	303.0 (8.1)	159.0 (10.7)	0.003
Chronic kidney disease	459.0 (8.8)	285.0 (7.7)	174.0 (11.8)	<0.001
Shock history	477.0 (9.2)	329.0 (8.8)	148.0 (10.0)	0.187
Liver disease	286.0 (5.5)	170.0 (4.6)	116.0 (7.8)	<0.001
Hypertension	457.0 (8.8)	300.0 (8.1)	157.0 (10.6)	0.003
Congestive heart failure	504.0 (9.7)	323.0 (8.7)	181.0 (12.2)	<0.001
Coronary heart disease	163.0 (3.1)	115.0 (3.1)	48.0 (3.2)	0.769
Malignant tumors	207.0 (4.0)	148.0 (4.0)	59.0 (4.0)	0.981
Diabetes	934.0 (17.9)	641.0 (17.2)	293.0 (19.8)	0.028
Severity score^a^				
APACHE IV	66.0 (52.0, 84.0)	63.0 (50.0, 78.0)	76.0 (60.0, 95.0)	<0.001
SOFA	6.0 (4.0, 9.0)	6.0 (4.0, 8.0)	8.0 (6.0, 10.0)	<0.001
GCS	15.0 (12.0, 15.0)	15.0 (13.0, 15.0)	14.0 (10.0, 15.0)	<0.001
LOS before admission to ICU, days	1.3 (0.1, 0.4)	1.2 (0.1, 0.4)	1.6 (0.0, 0.6)	0.663
Vital signs^b^				
Maximum heart rates (beat/min)	113.0 (98.0, 128.0)	112.0 (98.0, 126.0)	116.0 (100.0, 133.0)	<0.001
Minimum heart rates (beat/min)	76.0 (66.0, 88.0)	76.0 (66.0, 87.0)	78.0 (66.0, 90.0)	0.001
Maximum respiratory rates (time/min)	30.0 (26.0, 36.0)	30.0 (25.0, 36.0)	31.0 (26.0, 38.0)	<0.001
Minimum respiratory rates (time/min)	14.0 (11.0, 16.0)	14.0 (11.0, 16.0)	14.0 (11.0, 17.0)	0.733
Maximum mean arterial pressure (mmHg)	102.0 (90.0, 117.0)	102 (90.0, 116.0)	104.0 (90.0, 117.0)	<0.001
Minimum mean arterial pressure (mmHg)	54.0 (46.0, 63.0)	55.0 (47.0, 63.0)	53.0 (44.0, 60.0)	<0.001
Maximum temperature (°C)	38.0 (37.1, 38.5)	38.0 (37.1, 38.4)	38.0 (37.1, 36.8)	<0.001
Minimum temperature (°C)	36.0 (36.1, 36.7)	36.0 (36.0, 36.8)	36.0 (36.0, 36.7)	0.889
Laboratory results^c^				
Maximum sodium (mmol/L)	139.0 (136.0, 142.0)	139.0 (136.0, 142.0)	139.0 (136.0, 143.0)	0.002
Minimum sodium (mmol/L)	136.0 (133.0, 139.0)	136.0 (133.0, 139.0)	136.0 (133.0, 139.0)	0.135
Maximum potassium (mmol/L)	4.4 (4.0, 5.0)	4.4 (3.9, 4.9)	4.4 (4.0, 5.1)	0.002
Minimum potassium (mmol/L)	3.8 (3.4, 4.3)	3.9 (3.5, 4.3)	3.8 (3.4, 4.3)	0.404
Maximum hemoglobin (g/dL)	11.4 (9.8, 13.1)	11.5 (9.9, 13.1)	11.2 (9.7, 13.0)	0.046
Minimum hemoglobin (g/dL)	10.1 (8.7, 11.7)	10 (8.5, 11.6)	10.2 (8.7, 11.7)	0.030
Maximum calcium (mg/dL)	8.6 (8.0, 9.1)	8.6 (8.0, 9.1)	8.5 (7.9, 9.0)	<0.001
Minimum calcium (mg/dL)	7.9 (7.4, 8.4)	7.8 (7.4, 8.4)	7.9 (7.3, 8.4)	<0.001
Maximum RDW (%)	16.0 (14.5, 17.4)	16.0 (14.5, 17.2)	16.0 (14.8 ,17.8)	<0.001
Minimum RDW (%)	16.1 (14.3, 17.0)	15.0 (14.2, 16.9)	16.1 (14.5, 17.0)	<0.001
Maximum white blood cell (×10^3^/μL)	17.0 (13.0, 24.0)	16.0 (11.0, 22.0)	20.0 (15.0, 25.0)	<0.001
Minimum white blood cell (×10^3^/μL)	8.0 (5.0, 10.0)	8.0 (5.0, 10.0)	7.0 (5.0, 10.0)	<0.001
Maximum creatinine (mg/dL)	1.8 (1.2, 3.0)	2.0 (1.1, 2.9)	1.7 (1.2, 3.3)	<0.001
Minimum creatinine (mg/dL)	1.4 (0.9, 2.4)	1.6 (0.9, 2.2)	1.4 (1, 2.6)	<0.001
Maximum lactate (mmol/L)	2.5 (1.6, 3.7)	2.6 (1.6, 3.6)	2.5 (1.6, 3.9)	<0.001
Minimum lactate (mmol/L)	1.7 (1.1, 2.3)	1.7 (1.1, 2.2)	1.7 (1.2, 2.3)	0.001
Maximum BUN (mg/dL)	34.0 (21.0, 52.0)	32.0 (21.0, 50.0)	37.0 (22.0, 56.0)	<0.001
Minimum BUN (mg/dL)	27.0 (17.0, 43.0)	31.0 (17.0, 42.0)	26.0 (18.0, 47.0)	<0.001
Maximum albumin (mmol/L)	3.1 (2.5, 3.4)	3.1 (2.5, 3.5)	3.0 (2.4, 3.3)	<0.001
Minimum albumin (mmol/L)	2, 6 (2.2, 3.0)	2, 6 (2.3, 3.1)	2.5 (2.1, 2.9)	<0.001
Maximum RBC (m/μL)	3.9 (3.3, 4.4)	3.9 (3.3, 4.4)	3.9 (3.3, 4.4)	0.098
Minimum RBC (m/μL)	3.5 (3.0, 4.0)	3.5 (3.0, 4.0)	3.5 (3.0, 4.0)	0.209
Maximum platelet (×10^3^/μL)	209.0 (148.0, 281.0)	209.0 (149.0, 280.0)	209.0 (144.0, 238.0)	0.681
Minimum platelet (×10^3^/μL)	177.0 (122.0, 238.0)	177.0 (124.0, 239.0)	175.0 (118.0, 238.0)	0.315
Maximum MCV (fl)	91.0 (87.0, 96.0)	91.0 (87.0, 95.0)	91.0 (87.0, 96.0)	0.051
Minimum MCV (fl)	90.0 (85.0, 94.0)	90.0 (85.0, 94.0)	90.0 (85.0, 94.0)	0.355
Maximum glucose (mg/dL)	180.0 (137.0, 247.0)	177.0 (136.0, 246.0)	187.0 (140.0, 251.0)	0.004
Minimum glucose (mg/dL)	106.0 (86.0, 133.0)	106.0 (86.0, 136.0)	107.0 (86.0, 134.0)	0.362
Maximum bilirubin (umol/L)	0.9 (0.5, 1.5)	0.9 (0.5, 1.5)	0.9 (0.5, 1.6)	0.027
Minimum bilirubin (umol/L)	0.8 (0.5, 1.3)	0.8 (0.4, 1.2)	0.8 (0.5, 1.3)	0.027
Maximum bicarbonate (mmol/L)	24.0 (21.0, 27.0)	24.0 (22.0, 27.0)	25.0 (21.0, 28.0)	0.389
Minimum bicarbonate (mmol/L)	22.0 (18.0, 25.0)	21.0 (18.0, 25.0)	22.0 (18.0, 25.0)	0.031
Maximum chloride (mmol/L)	105.0 (101.0, 109.0)	105.0 (101.0, 109.0)	104.0 (100.0, 109.0)	0.540
Minimum chloride (mmol/L)	100.0 (96.0, 105.0)	101.0 (96.0, 105.0)	100.0 (96.0, 105.0)	0.926
Infection sites, *n* (%)				<0.001
Pulmonary	1,835.0 (35.3)	1,180.0 (31.7)	625.0 (44.3)	
Renal	598.0 (11.5)	423.0 (11.4)	175.0 (11.8)	<0.001
Gastrointestinal tract	1,259.0 (24.2)	1,006.0 (27.0)	253.0 (17.1)	<0.001
Skin/soft tissue	576.0 (11.06)	415.0 (11.14)	161.0 (10.88)	<0.001
Others/Unknown	938.0 (18.2)	702.0 (18.8)	236.0 (16.0)	<0.001
Interventions (1st 24 h), *n* (%)				
Ventilation, *n* (%)	2,468.0 (47.4)	1,506.0 (40.4)	962.0 (65.0)	<0.001
Vasopressors, *n* (%)	1,487.0 (28.6)	957.0 (25.7)	530.0 (35.8)	<0.001
Dialysis, *n* (%)	342.0 (6.6)	217.0 (5.8)	125.0 (8.5)	<0.001
Clinical outcomes				
ICU LOS, days	3.0 (1.8, 5.5)	2.2 (1.7, 3.2)	8.1 (6.2, 12.0)	<0.001
Hospital LOS, days	7.3 (4.6, 12.2)	5.8 (3.8, 8.8)	13.4 (9.1, 19.6)	<0.001
ICU mortality, *n* (%)	456.0 (8.8)	268.0 (7.2)	188.0 (12.7)	<0.001
Hospital mortality, *n* (%)	726.0 (14.0)	434.0 (11.7)	292.0 (19.7)	<0.001

Continuous data are presented as median (interquartile range), whereas categorical data are presented as frequency (percentage). Data are expressed as median (IQR), or n (%). Analysis of variance (or the Kruskal-Wallis test) and Chi-square (or Fisher's exact) tests were used for comparisons among groups. Statistical significance (P < 0.05).

a Severe score is calculated on the first day of each ICU patients' stay.

b Vital signs are calculated on the first 24 h of each ICU patients' stay.

c Laboratory results the first result of each patients' ICU stay.

APACHE IV, acute physiology and chronic health evaluation IV; SOFA, sequential organ failure assessment; GCS, glasgow coma scale; RDW, red cell volume distribution width; RBC, red blood cell; MCV, mean red cell volume; BUN, blood urea nitrogen; ICU, intensive care unit; p-LOS, prolonged length of stay.

### ICU p-LOS was a risk factor of ICU mortality in obesity patients with sepsis

After adjusting for baseline characteristics, vital signs, laboratory tests, and infection site, the results of the univariate and multivariate logistic regressions revealed that ICU p-LOS was an independent risk factor for ICU mortality of the participants [odds ratio (OR) = 1.88, 95% confidence interval (CI) 1.54–2.29, *p* < 0.001; OR = 1.39, 95% CI 1.08–1.79, *p* = 0.012; respectively] (Table 3). Following PSM between ICU p-LOS and non-ICU p-LOS groups based on differences in baseline characteristics, vital signs, laboratory results, and infection sites (Supplementary Table S2), results of univariate and multivariate logistic regression after adjusting for baseline characteristics, vital signs, laboratory results, and infection sites revealed that ICU p-LOS was an independently risk factor for ICU mortality of the participants [OR = 1.34, 95% CI 1.03–1.73, *p* = 0.027; OR = 1.41, 95% CI 1.04–1.91, *p* = 0.026; respectively] (Table 3). Above all, the results showed that whether PSM was used or not, there were significant differences in ICU mortality between the ICU p-LOS and non-ICU p-LOS groups. Therefore, constructing a comprehensive nomogram provides the accurate and straightforward personalized prediction of ICU p-LOS with clinical utility.

**Table 3:** Correlation of ICU p-LOS with ICU mortality of obesity patients with sepsis in the original and post-PSM cohort.

	**Original cohort**				**Post-PSM cohort**		
**Variables**	**OR**	**95% CI**	***P*** **-value**	**Variables**	**OR**	**95% CI**	***P*** **-value**
**Univariate logistic regression**							
ICU p-LOS	1.878	1.542–2.287	<0.001	ICU p-LOS	1.336	1.033–1.728	0.027
**Multivariate logistic regression**							
ICU p-LOS	1.388	1.075–1.791	0.012	ICU p-LOS	1.411	1.042–1.911	0.026
Pulmonary	1.583	1.070–2.342	0.021	APACHE IV	1.022	1.015–1.030	<0.001
APACHE IV	1.017	1.011–1.023	<0.001	SOFA	1.192	1.116–1.274	<0.001
SOFA	1.202	1.143–1.265	<0.001	GCS	1.089	1.034–1.147	0.001
GCS	1.101	1.054–1.149	<0.001	LOS before admission to ICU	1.047	1.023–1.071	<0.001
LOS before admission to ICU	1.056	1.036–1.077	<0.001	Minimum white blood cell	1.105	1.063–1.149	<0.001
Congestive heart failure	1.522	1.064–2.177	0.022	Maximum bicarbonate	1.065	1.007–1.126	0.028
Ventilation	1.445	1.104–1.892	0.007				
Vasopressors	1.288	1.002–1.656	0.048				
Minimum mean arterial pressure	0.989	0.980–0.998	0.013				
Minimum platelet	0.996	0.992–1.000	0.030				
Minimum white blood cell	1.118	1.090–1.147	<0.001				
Minimum lactate	1.134	1.028–1.251	0.012				
Minimum albumin	0.886	0.792–0.992	0.036				

PSM, propensity score matching; OR, odds ratio; CI, confidence interval; APACHE IV, acute physiology and chronic health evaluation IV; SOFA, sequential organ failure assessment; GCS, Glasgow coma scale; ICU, intensive care unit; p-LOS, prolonged length of stay.

### Development of a prediction nomogram

**Figure 2:**
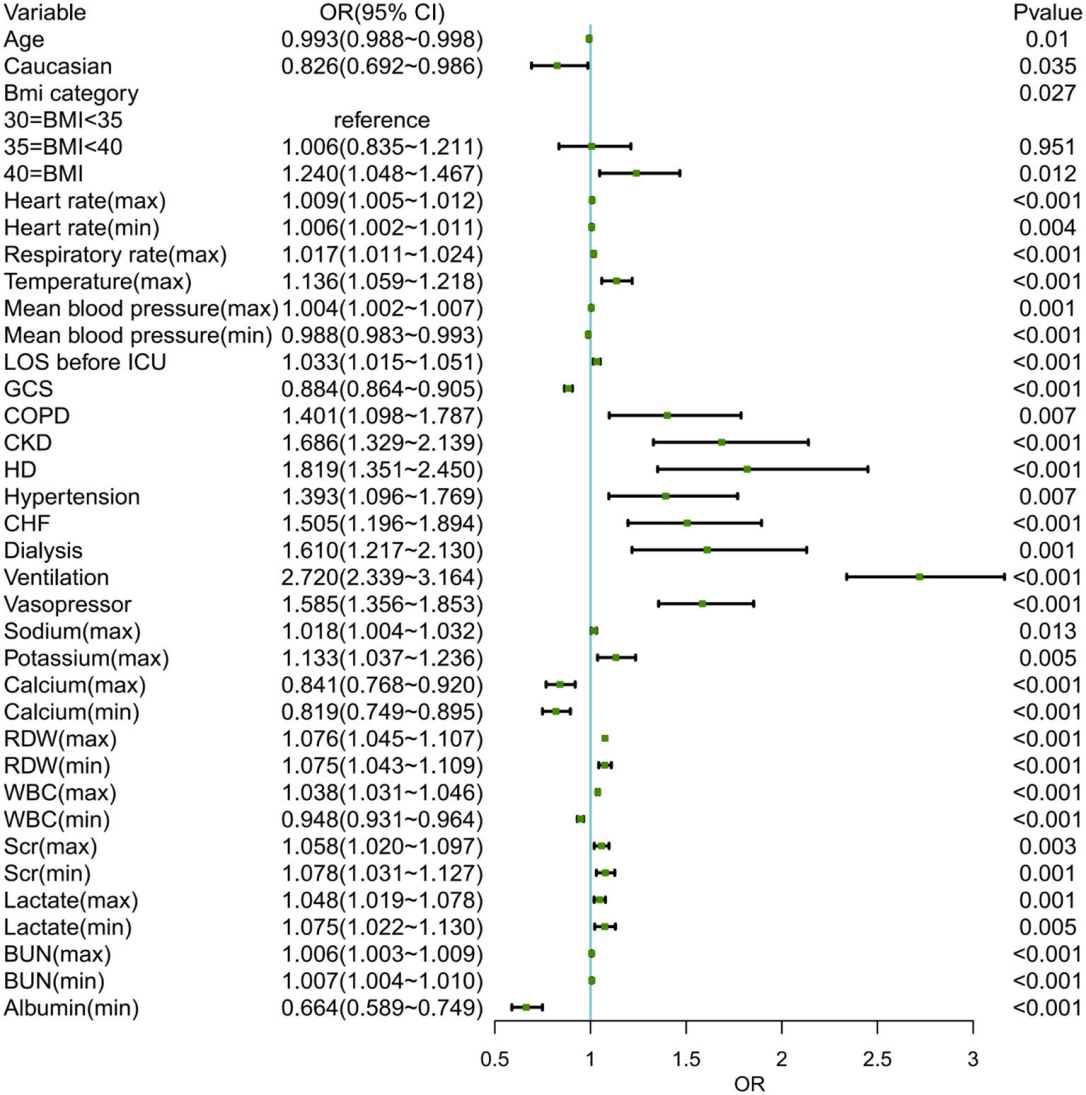
The univariate logistic analysis of obese patients with sepsis in the training set. BMI, body mass index; LOS, length of stay; ICU, intensive care unit; GCS, Glasgow coma scale; COPD, chronic obstructive pulmonary diseases; CKD, chronic kidney disease; LD, liver disease; CHF, congestive heart failure; RDW, red cell volume distribution width; WBC, white blood cell; BUN, blood urea nitrogen.

The training set (3,606 patients) and internal validation set (1,600) of 5,206 obesity patients with sepsis were assigned at random. The model was created to predict the likelihood of ICU p-LOS in obesity patients with sepsis. In Supplementary Table S3, all variables of the patients in each set were listed. The results revealed that there was no statistical difference between the two groups in all variables. Figure 2 showed the results of the univariate logistic analysis using the training set.

**Figure 3:**
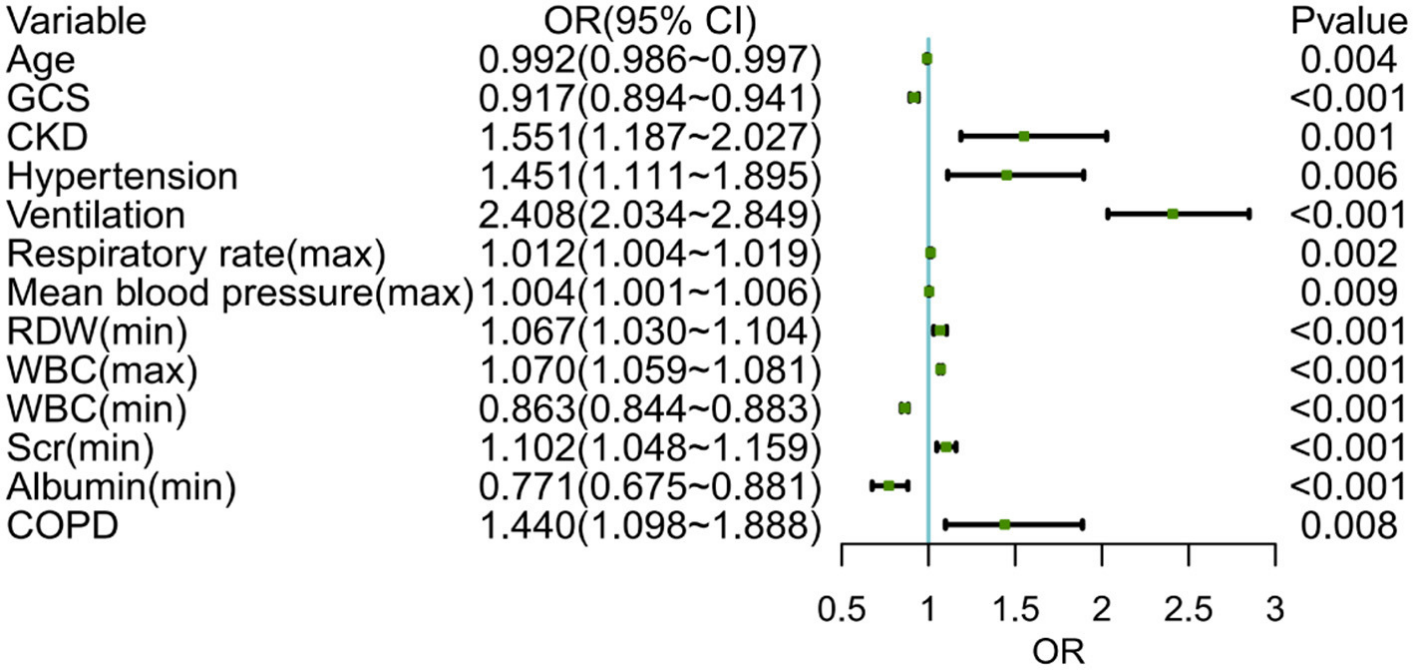
The multivariable logistic regression of obese patients with sepsis in the training set. GCS, Glasgow coma scale; CKD, chronic kidney disease; RDW, red cell volume distribution width; WBC, white blood cell; COPD, chronic obstructive pulmonary diseases.

**Figure 4:**
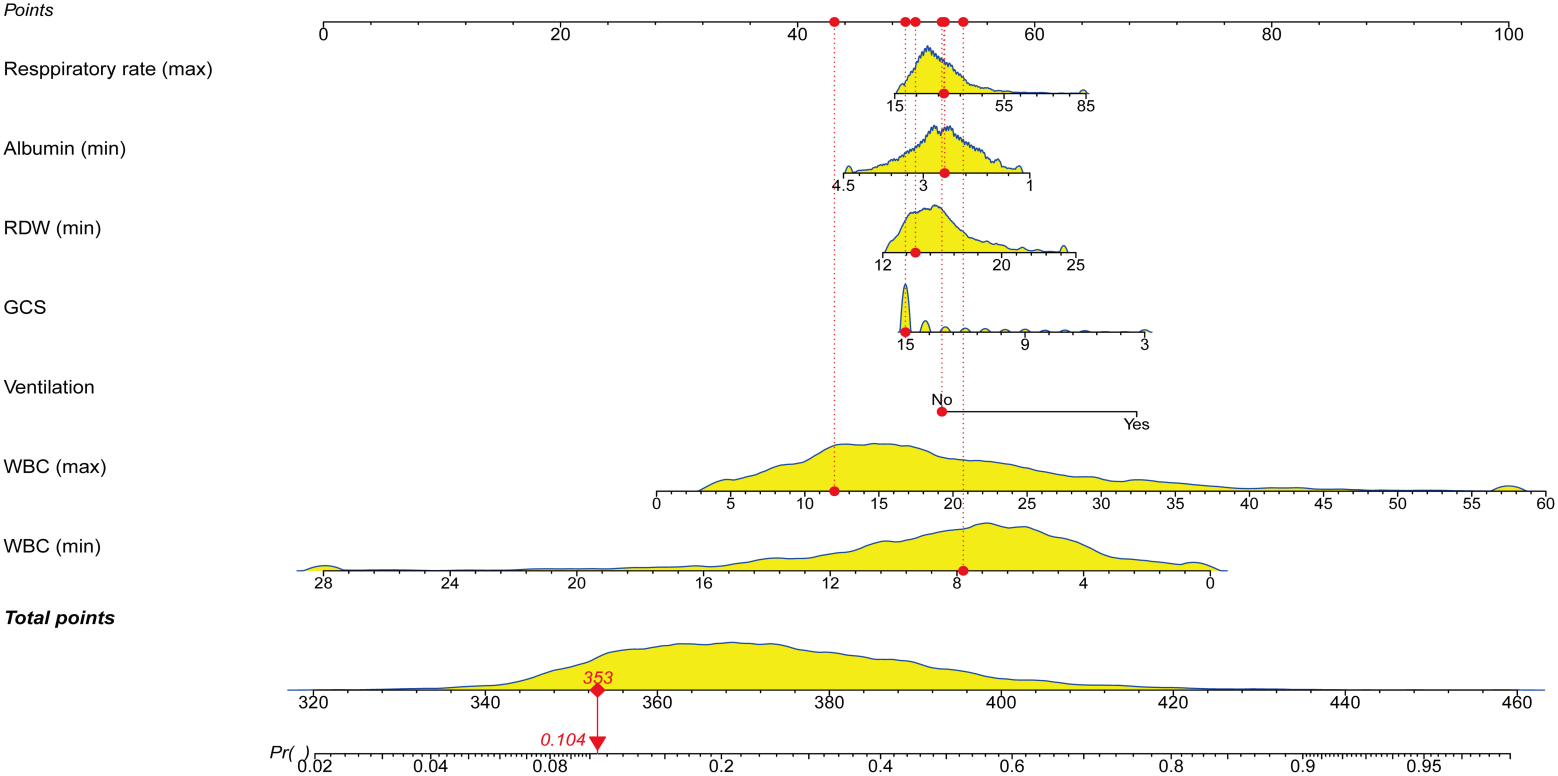
Nomogram for predicting the probability of ICU p-LOS in obese patients with sepsis. RDW, red cell distribution width; GCS, Glasgow coma scale; WBC, white blood cell; ICU, intensive care unit; p-LOSz, prolonged length of stay.

Following that, we used variables with *p* < 0.05 in the univariable logistic analysis, those with clinical significance, or categorical variables with a set of meaningful values in a multivariate logistic regression. Figure 3 showed the risk factors identified by multivariable logistic regression that were independently related to ICU p-LOS of obesity patients with sepsis. Regarding the collinearity of the variables, the VIF was calculated and visualized in Supplementary Figure S1. The result was <2, which means no collinearity in the regression analysis. Then we calculated the relative importance of predictor variables (Supplementary Figure S2). According to the Occam's Law of Razor (41), we excluded chronic kidney disease, minimum serum creatinine, maximum mean blood pressure, age, hypertension, and chronic obstructive pulmonary disease, since they only explained <10% of Logistic regression cumulative deviance explained. Finally, a model integrating maximum WBC, minimum WBC, use of ventilation, GCS, minimum albumin, maximum respiratory rate, and minimum RDW was established. The Hosmer-Lemeshow test yielded a *P*-value of 0.432, indicating that the model was well fitted. A nomogram was also plotted based on this model to predict the probability of ICU p-LOS in obesity patients with sepsis (Figure 4).

### Validation of the prediction nomogram

Our external validation set, derived from the MIMIC IV database, was based on similar inclusion criteria and extracted relevant seven variables, excluding the samples with missing values, and 2,424 samples were finally obtained. In the external validation set, the third quartile value of ICU LOS was about 6 days, so patients with an ICU LOS of 6 days or more were considered to have ICU p-LOS in the external validation set.

**Figure 5:**
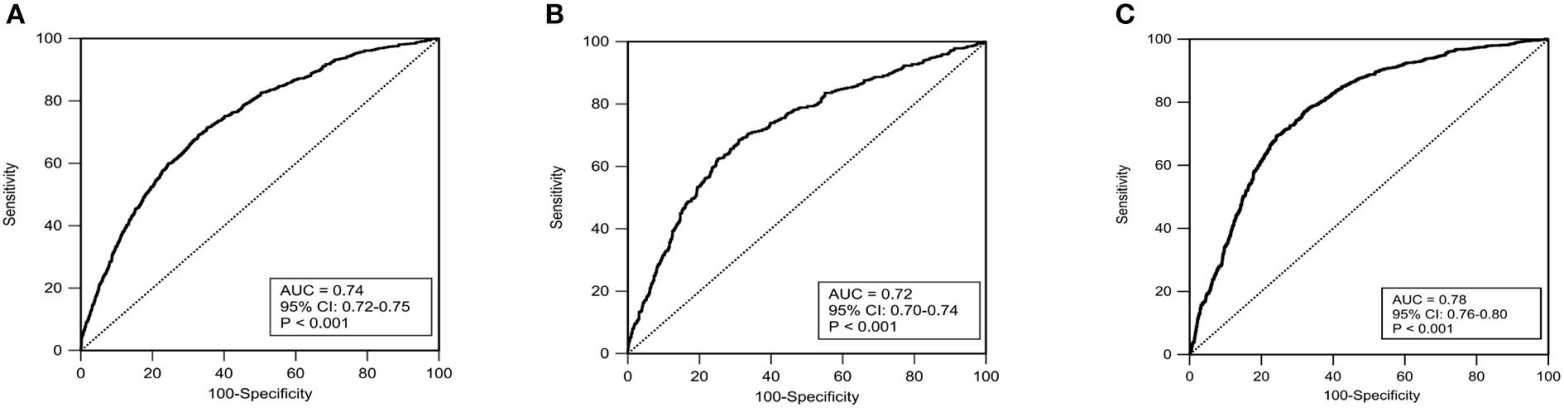
The receiver operating characteristic curves (ROC) in the training set **(A)**, internal validation set **(B)** and external validation set **(C)**. AUC, area under curve; Cl, confidence interval.

The ROC curves in Figures 5A–C showed that our nomogram not only had an excellent discriminative ability in internal validation set (AUC = 0.73, 95% CI 0.71–0.75), but also a good discriminative ability in external validation (AUC = 0.78, 95% CI 0.76–0.80).

For ICU performance benchmarking and quality improvement analysis, APACHE IV was used to risk-adjust ICU patients. Besides, APACHE IV had been validated for predicting ICU LOS (38, 39). SOFA, like APACHE IV, was also shown to be a predictor of ICU LOS alone (43). By comparing their AUC and IDI to judge the performance of nomogram, we found that the discrimination performance of nomogram for ICU p-LOS was significantly better than APACHE IV and SOFA (*P* < 0.001), our nomogram improved the performance of them by about 10–15% (Table 4).

**Table 4:** Comparison of models in predicting the probability of ICU p-LOS of obesity patients with sepsis.

**Predictive model**		**AUROC**	***P*** **-value**	**IDI**	***P*** **-value**
Training set	APACHE IV	0.647 (0.631–0.663)	<0.001	0.099 (0.087–0.112)	<0.001
	SOFA	0.670 (0.654–0.685)	<0.001	0.083 (0.070–0.096)	<0.001
	Nomogram	0.742 (0.727–0.756)			
Internal validation set	APACHE IV	0.655 (0.631–0.678)	<0.001	0.085 (0.067–0.103)	<0.001
	SOFA	0.648 (0.624–0.671)	<0.001	0.089 (0.071–0.107)	<0.001
	Nomogram	0.730 (0.708–0.752)			
External validation set	APACHE IV	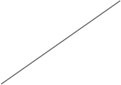	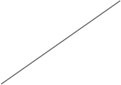	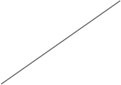	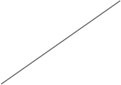
	SOFA	0.558 (0.535–0.581)	<0.001	0.196 (0.180–0.212)	<0.001
	Nomogram	0.778 (0.760–0.797)			

AUROC, area under the receiver operating characteristic curve; CI, confidence interval; IDI, integrated discrimination improvement; APACHE IV, acute physiology and chronic health evaluation IV; ICU, intensive care unit; p-LOS, prolonged length of stay.

**Figure 6:**
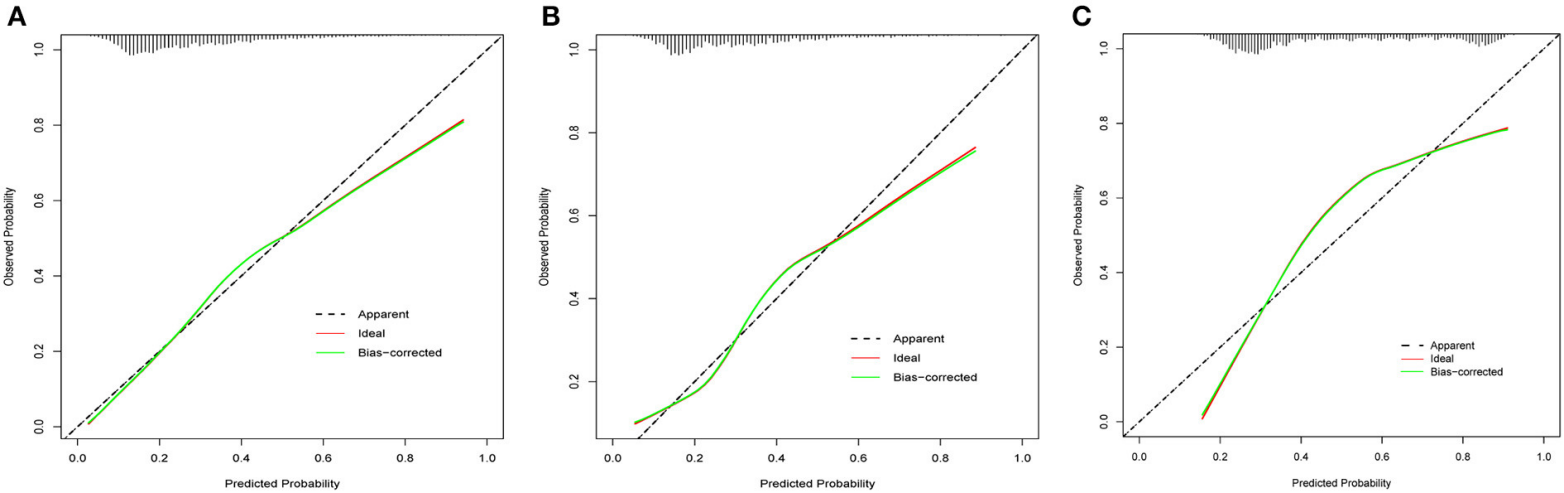
The calibration curves for the nomogram in the training set **(A)**, internal validation set **(B)** and external validation set **(C)**.

For all of the training, internal validation, and external validation sets, the calibration curves were described using the bootstrap method. In all sets, the apparent curve and bias-corrected curve deviated slightly from the reference line, but there was still good agreement between observation and prediction. Figures 6A–C depicted the details. Furthermore, the nomogram's Brier scores in the training set were 0.172, 0.178 in the internal validation set, and 0.197 in the external validation set, indicating that the nomogram's prediction calibration was good.

### Clinical use of the nomogram

**Figure 7:**
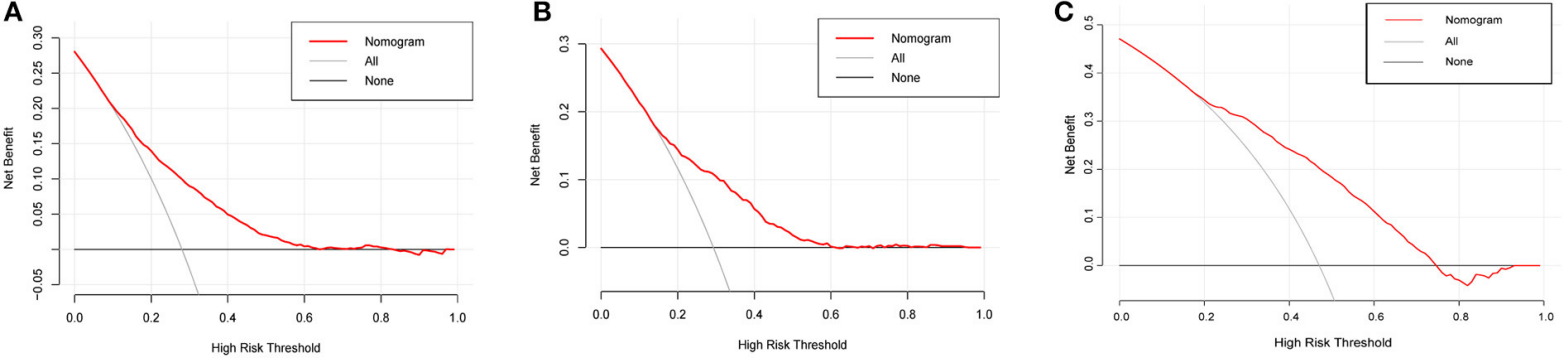
The decision curves analysis (DCA) for the nomogram in the training set **(A)**, internal validation set **(B)** and external validation set **(C)**.

**Figure 8:**
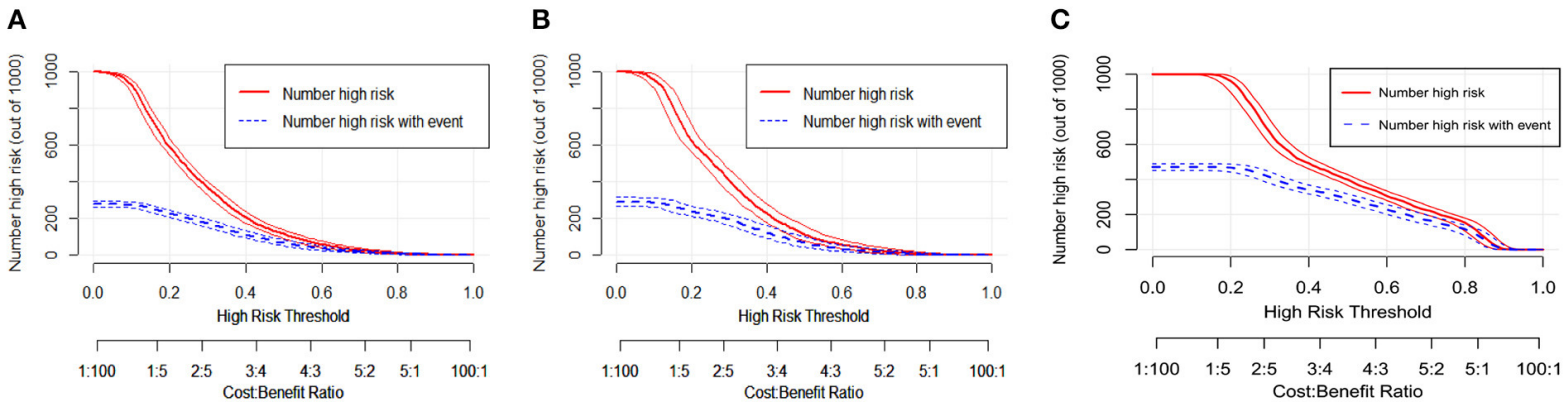
The clinical impact curves (CIC) for the nomogram in the training set **(A)**, internal validation set **(B)** and external validation set **(C)**.

To perform a clinical application of this nomogram, we plotted the DCA curve. In three sets of circumstances, medical intervention guided by this nomogram could provide an excellent net benefit. Figures 7A–C depicted the details. We presented the nomogram's clinical impact curve (CIC) based on the DCA. Figures 8A–C depicts the outcomes. The solid red curve (number of high-risk individuals) represented the number of patients classified as positive (high risk) by the nomogram at each 1,000-patient threshold, while the dotted blue curve (number of true positive patients) represented the number of true positive patients under each risk threshold. CIC confirmed the clinical value of the nomogram by visually indicating that it provided a high clinical net benefit. Besides, to facilitate convenient clinical use, an online dynamic nomogram (https://cy19940626.shinyapps.io/DynNomapp/) based on this model was built.

### Risk of ICU p-LOS based on the nomogram scores

The nomogram was found to be a useful predictive model with high sensitivity, specificity, positive predictive value, and negative predictive value in determining whether the ICU LOS of obesity patients with sepsis was relatively prolong, with 0.68 (95% CI: 0.65, 0.71), 0.69 (95% CI: 0.67, 0.71), 0.46 (95% CI: 0.44, 0.50) and 0.85 (95% CI: 0.83, 0.86) in the training set; 0.70 (95% CI: 0.65, 0.74), 0.68 (95% CI: 0.65, 0.71), 0.47 (95% CI: 0.44, 0.53), and 0.84 (95% CI: 0.82, 0.86) in the internal validation set; 0.69 (95% CI: 0.67, 0.72), 0.76 (95% CI: 0.73, 0.78), 0.72 (95% CI: 0.69, 0.74), 0.74 (95% CI: 0.71, 0.96) in the external validation set; respectively.

## Discussion

In our retrospective overall sepsis cohort of the EICU database (14,483 sepsis patients), the obesity group had a longer ICU LOS than the other three non-obesity groups. Meanwhile, there was a trend of ICU p-LOS in the obesity group, which was consistent with the conclusions of previous reports. Moreover, the findings revealed that the obesity group in our sepsis cohort had lower mortality than the other three non-obesity groups, providing new clinical evidence for the obesity paradox of sepsis.

In the obesity patients with sepsis cohort, we found that ICU p-LOS was an independent risk factor for ICU mortality regardless of PSM implementation. To identify the independent risk factor associated with the ICU p-LOS of obesity patients with sepsis in the ICU, we used univariate and multivariate logistic regression analyses. Finally, seven clinical variables were identified and incorporated into the best-fitting model, which was visualized as a prediction nomogram, that is, maximum WBC, minimum WBC, use of ventilation, GCS, minimum albumin, maximum respiratory rate, and minimum RDW. To our knowledge, this is the first study to examine the relationship between ICU p-LOS and ICU mortality in obesity patients with sepsis, as well as to identify relevant independent risk factors for ICU p-LOS and to develop a nomogram to predict it.

Among these seven included factors, the WBC count accounted for the most prominent weight in the nomogram. As we all recognize, the principal function of white blood cells is defense. Sepsis can be thought of as a death race between pathogens and the immune system of the host (3). An empirical model revealed that losing lymphocytes could lead to heightened mortality from sepsis. Patients with a higher NLCR had a higher white blood cell count, higher neutrophil count, lower lymphocyte count, and higher 28-day mortality or longer ICU LOS, according to a large cohort study (44). Another study found that monocyte PDL1 expression is an independent predictor of sepsis-related 28-day mortality in patients (45). Above all, we can conclude that WBC counts are considerably associated with poor prognosis in patients with sepsis or septicemia. The smaller the WBC count, the stronger the immunosuppressive response. Likewise, the conclusion was also applied to obesity patients. The innate immune system has been a primary element in established obesity (46). Furuncuoglu et al. and Maurizi et al. showed that BMI was significantly positively correlated with neutrophil, lymphocyte and WBC counts (47, 48). Several possible reasons could reveal the interrelationship between the WBC and adverse prognosis for sepsis or obesity patients, despite the immune mechanisms involved being unclear. Numerous studies have determined that many patients who died of sepsis or MODS had immunosuppressive features (49). The delayed apoptosis of neutrophils and the appearance of immature band-like neutrophils in peripheral blood with antimicrobial effector function deficits, including oxidative burst capacity, is a key finding in sepsis (50). Moreover, increased lymphocyte apoptosis in the thymus and spleen contributes to immunosuppression, sepsis and obesity (51, 52).

During sepsis, patients required invasive ventilation, and among these patients, supplemental oxygen was also essential (53). A single-center RCT pointed out that mechanical ventilation and usual oxygen therapy could have a clinically meaningful effect on the poor prognosis of patients. Meanwhile, they found that when compared to standard oxygen therapy, mechanical ventilation did not result in a statistically significant reduction in 90-day mortality (54). Currently, RDW is considered to be a crucial factor for human mortality in various diseases: hematological malignancies (55), cardiovascular diseases (56), and critical illness (57). RDW was associated with mortality in patients with sepsis, according to Zhang et al.'s findings, and it could be a useful and simple prognostic marker for patients with sepsis (58). Furthermore, RDW was found to be associated with traditional inflammatory biomarkers independently by Lippi et al. (59). In a recent cohort study that concluded RDW was more related to the prognosis of patients whose BMI was >25, there was also a significant interaction between RDW and BMI in terms of all-cause mortality (60). In addition, some vital signs and scores are used to predict sepsis. As demonstrated by Wijdicks et al., Glasgow Coma Scale score was an independent predictor of mortality in ICU patients (61). GCS and an abnormal respiratory rate, according to Lane et al., can identify patients with a higher morbidity and mortality of sepsis (62). In a randomized controlled trial, administration of albumin may have decreased the risk of death, which indicated it was associated with albumin with the prognostics of sepsis patients (63). In short, it strongly associated all seven of these inclusion factors in the nomogram with poor prognosis in sepsis or obesity patients.

Our nomogram is concise, practical containing only seven clinical variables. Based on the AUROC and calibration curve, it also shows acceptable discrimination and good calibration ability. It effectively calculates the risk probability of ICU p-LOS for obesity patients with sepsis; therefore, it provides early information on ICU p-LOS for sepsis with obesity patients admitted to the ICU and helps ICU clinicians to develop strategies and plans accordingly in advance. When considering that a particular sepsis with obesity patient is at high risk of ICU p-LOS (at least above 50%), on the one hand, there may be a possibility of shortening the ICU LOS by targeting the corresponding indicators based on the independent risk factors for ICU p-LOS in sepsis with obesity patients identified in this study, but further rigorous clinical studies are needed to verify the effectiveness. On the other hand, based on experience in the treatment and management of sepsis in the ICU, we recommend that (1) The ICU physician should develop a preferred empirical treatment strategy based on local patterns of resistance, the most prevalent pathogens associated with the known or suspected sites of sepsis infection, any host parameters associated with the risk of uncommon or drug-resistant pathogens, and considering both local and national guidelines and local antimicrobial sensitivities, and when a high risk of ICU p-LOS in sepsis with obesity patient is identified, initiate appropriate empirical antibiotic therapy to reduce ICU LOS and improve clinical outcomes in the ICU. (2) Gram staining and culture, molecular diagnostics and calcitoninogen monitoring are novel tools whose application has the potential to enhance the ability of clinical practitioners to administer antibiotics and can facilitate more effective, targeted antibiotic therapy for sepsis. (3) Therapeutic drug monitoring is a useful strategy to facilitate more accurate application of antibiotic doses to avoid under-dosing leading to possible antibiotic resistance, while encouraging clinicians to reduce the total duration of antibiotic therapy and lower antibiotic levels during a course of treatment. (4) Given the distribution of patients in the ICU, changes in elimination and the risk of resistance, an individual approach to dosing should also be adopted and, if possible, clinicians should consult the ICU pharmacist to further refine antibiotic regimens to sepsis.

Notwithstanding that it based our study on a combination of many ICUs over the entire mainland United States, it even retains several limitations. Firstly, our data were from the USA, therefore the results may not apply to ICUs in other countries. Second, this was a retrospective analysis in which recall bias was necessary. Thus, a prospective cohort study was required for further validation. Third, as seen in most of previous studies, the absolute days of stay in ICU varied from one hospital to another. Although our nomogram demonstrated a promising prediction capability within the internal eICU-CRD and MIMIC IV. However, scalability to other hospitals remains an issue and needs to be used with caution. Therefore, when other hospitals would apply this nomogram, they should start by surveying the ICU LOS across the institution and calculating the third quartile of sepsis pat to determine their own LOS prolongation threshold. Fourth, BMI is not the only way to define obesity, other indicators that can be used to define obesity include: waist to hip ratio, visceral obesity index and waist to height ratio. However, as this is a retrospective study with data from EICU and MIMIC IV, the lack of these indicators leaves us with BMI as the only way to define obesity. Therefore, there is a need for a prospective study in the future to define obesity by various indicators in order to study the role of obesity in sepsis or other diseases, which may lead to some new findings. To sum up, our nomogram is quite promising and worthy of further exploration in future clinical work and research.

## Conclusion

We investigated specific predictors of ICU p-LOS in obesity patients with sepsis. We constructed a new nomogram to predict the risk of ICU p-LOS in obesity patients with sepsis using seven risk factors (maximum WBC, minimum WBC, use of ventilation, GCS, minimum albumin, maximum respiratory rate, and minimum RDW). This prediction model is accurate and reliable, and it can assist patients and clinicians in determining prognosis and clinical decisions.

## Data availability statement

The raw data supporting the conclusions of this article will be made available by the authors, without undue reservation.

## Ethics statement

Ethical review and approval was not required for the study on human participants in accordance with the local legislation and institutional requirements. Written informed consent for participation was not required for this study in accordance with the national legislation and the institutional requirements.

## Author contributions

Research was designed by YaC, ML, YH, and QH. ML and YaC conducted experiments and analyzed data. The main manuscript text and figures were written by YaC, ML, YH, and YuC. The manuscript was edited and revised by YaC and QH. All authors contributed to the article and gave their approval to the final version.

## Funding

China Education and Research Network (NGII20190706 and NGII20180703) funded this study.

## Conflict of interest

The authors declare that the research was conducted in the absence of any commercial or financial relationships that could be construed as a potential conflict of interest.

## Publisher's note

All claims expressed in this article are solely those of the authors and do not necessarily represent those of their affiliated organizations, or those of the publisher, the editors and the reviewers. Any product that may be evaluated in this article, or claim that may be made by its manufacturer, is not guaranteed or endorsed by the publisher.
