# Supplementary material for: A nomogram to predict prolonged stay of obesity patients with sepsis in ICU: Relevancy for predictive, personalized, preventive, and participatory healthcare strategies
Source: Front Public Health. 2022 Aug 11;10:944790. doi: 10.3389/fpubh.2022.944790 (PMC9403617; doi:10.3389/fpubh.2022.944790)
Supplement: Supplementary file 1 [file Table_1.DOCX]

| **Supplementary Table S1. Comparison of ICU LOS among four groups classified according to BMI.** | | | | | | | | | | | | | | | | |
| --- | --- | --- | --- | --- | --- | --- | --- | --- | --- | --- | --- | --- | --- | --- | --- | --- |
| One-Way ANOVA Analysis | | | | | | | | | | | | | | | | |
| Test of Homogeneity of Variances | | | | | | | | | | | | | | | | |
|  | | | Levene Statistic | | | | | df1 | | df2 | | | | p-value | | |
| Based on Mean | | | 6.51 | | | | | 3 | | 14479 | | | | <0.001 | | |
| Based on Median | | | 4.14 | | | | | 3 | | 14479 | | | | 0.006 | | |
| Based on Median and with adjusted df | | | 4.14 | | | | | 3 | | 13165.85 | | | | 0.006 | | |
| Based on trimmed mean | | | 5.32 | | | | | 3 | | 14479 | | | | 0.001 | | |
|  | | | | | | | | | | | | | | | | |
| ANOVA | | | | | | | | | | | | | | | | |
|  | | Sum of Squares | | df | | | Mean Square | | | | F | | | | p-value | |
| Between Groups | | 583.61 | | 3 | | | 194.54 | | | | 5.49 | | | | 0.001 | |
| Within Groups | | 512870.40 | | 14479 | | | 35.42 | | | |  | | | |  | |
| Total | | 513454.01 | | 14482 | | |  | | | |  | | | |  | |
|  | | | | | | | | | | | | | | | | |
| Robust Tests of Equality of Means | | | | | | | | | | | | | | | | |
|  | | Statistic^a^ | | | | df1 | | | df2 | | | | p-value | | | |
| Welch | | 5.89 | | | | 3 | | | 3849.98 | | | | 0.001 | | | |
|  | | | | | | | | | | | | | | | | |
| Multiple Comparisons | | | | | | | | | | | | | | | | |
| LSD | (I) BMI group | (J) BMI group | | | Mean Difference (I-J) | | | Std. Error | | | p-value | 95% Confidence Internal | | | | |
|  |  |  |  |  |  |  |  |  |  |  |  | Lower Bound | | | | Upper Bound |
|  | Obesity group | Underweight group | | | 0.66916* | | | 0.22279 | | | 0.003 | 0.2325 | | | | 1.1059 |
|  |  | Normal weight group | | | 0.38638* | | | 0.12014 | | | 0.001 | 0.1509 | | | | 0.6219 |
|  |  | Overweight group | | | 0.33357* | | | 0.12691 | | | 0.009 | 0.0848 | | | | 0.5823 |

Notes: Statistical significance (P<0.05).

^a^. Asymptotically F distributed.

*. The mean difference is significant at the 0.05 level.

Abbreviations: ICU, intensive care unit; LOS, length of stay; BMI, body mass Index.
