# Supplementary material for: A nomogram to predict prolonged stay of obesity patients with sepsis in ICU: Relevancy for predictive, personalized, preventive, and participatory healthcare strategies
Source: Front Public Health. 2022 Aug 11;10:944790. doi: 10.3389/fpubh.2022.944790 (PMC9403617; doi:10.3389/fpubh.2022.944790)
Supplement: Supplementary file 2 [file Table_2.DOCX]

**Supplementary Table S2. Patients’ baseline characteristics in the post-PSM cohort (n=2478).**

| Characteristics | Total | Non-ICU p-LOS,  n=1239 | ICU p-LOS,  n=1239 | P-value |
| --- | --- | --- | --- | --- |
| Age, years | 64.0(54.0,73.0) | 64.0(54.0,73.0) | 65.0(55.0,73.0) | 0.308 |
| Male, n (%) | 1153.0(46.5) | 575.0(46.4) | 578.0(46.7) | 0.904 |
| Race, n (%) |  |  |  | 0.780 |
| Caucasian | 1928.0(77.8) | 957.0(77.2) | 971.0(78.4) |  |
| American | 304.0(12.3) | 157.0(12.7) | 147.0(11.9) |  |
| Other/Unknown | 246.0(9.9) | 125.0(10.1) | 121.0(9.8) |  |
| BMI, kg/m^2^ | 36.0(32.5,41.9) | 36.0(32.6,42.3) | 36.0(33.0,41.4) | 0.207 |
| LOS before admission to ICU, days | 1.5(0.1,0.5) | 1.5(0.1,0.5) | 1.5(0.1,0.6) | 0.706 |
| Comorbidities, n (%) |  |  |  |  |
| Chronic obstructive pulmonary diseases | 260.0(10.5) | 126.0(10.2) | 134.0(10.8) | 0.600 |
| Chronic kidney disease | 242.0(9.8) | 114.0(9.2) | 128.0(10.3) | 0.343 |
| Liver disease | 165.0(6.7) | 73.0(5.9) | 92.0(7.4) | 0.126 |
| Hypertension | 259.0(10.5) | 137.0(11.1) | 122.0(9.9) | 0.325 |
| Congestive heart failure | 280.0(11.3) | 134.0(10.8) | 146.0(11.8) | 0.446 |
| Shock history | 246.0(9.9) | 125.0(10.1) | 121.0(9.8) | 0.788 |
| Coronary heart disease | 84.0(3.4) | 39.0(3.2) | 45.0(3.6) | 0.505 |
| Malignant tumors | 101.0(4.0) | 51.0(4.1) | 50.0(4.0) | 0.911 |
| Diabetes | 498.0(20.1) | 260.0(20.0) | 238.0(19.2) | 0.270 |
| Severity score**^a^** |  |  |  |  |
| APACHE IV | 71.0(56.0,89.0) | 69.0(55.0,56.0) | 73.0(58.0,90.0) | <0.001 |
| SOFA | 7.0(5.0,10.0) | 7.0(5.0,10.0) | 7.0(5.0,10.0) | 0.029 |
| GCS | 14.0(11.0,15.0) | 14.0(11.0,15.0) | 14.0(11.0,15.0) | 0.818 |
| Vital Signs**^b^** |  |  |  |  |
| Maximum heart rates (beat/min) | 115.0(101.0,130.0) | 116.0(102.0.130.0) | 115.0(100.0,131.0) | 0.117 |
| Minimum heart rates (beat/min) | 79.0(68.0,90.0) | 80.0(70.0,91.0) | 77.0(65.5,89.0) | <0.001 |
| Maximum respiratory rates (time/min) | 31.0(26.0,38.0) | 31.0(26.0,37.6) | 31.0(26.0,38.0) | 0.715 |
| Minimum respiratory rates (time/min) | 14.0(11.0,17.0) | 14.0(11.0,17.0) | 13.0(11.0,17.0) | 0.010 |
| Maximum mean arterial pressure (mmHg) | 103.0(91.0.118.0) | 102.1(80.0,117.0) | 104.0(91.0,119.0) | 0.267 |
| Minimum mean arterial pressure (mmHg) | 54.0(46.0,62.0) | 55.0(46.0,63.0) | 53.2(45.0,61.0) | 0.015 |
| Maximum temperature (◦C) | 37.9(37.2,38.8) | 38.1(37.4,39.0) | 37.7(37.1.38.5) | <0.001 |
| Minimum temperature (◦C) | 36.6(36.2,36.9) | 36.8(36.5,37.1) | 36.4(36.0,36.9) | <0.001 |
| Laboratory results**^c^** |  |  |  |  |
| Maximum sodium (mmol/L) | 139.0(136.0,142.0) | 139.0(136.0,142.0) | 139.0(136.0,142.0) | 0.457 |
| Minimum sodium (mmol/L) | 136.0(133.0,139.0) | 136.0(133.0,139.0) | 136.0(133.0,139.0) | 0.522 |
| Maximum potassium (mmol/L) | 4.4(4.0,5.0) | 4.4(4.0,5.0) | 4.4(4.0,5.0) | 0.022 |
| Minimum potassium(mmol/L) | 3.8(3.4,4.3) | 3.8(3.4,4.3) | 3.8(3.4,4.3) | 0.025 |
| Maximum calcium (mmol/L) | 8.5(8.0,9.1) | 8.5(8.0,9.1) | 8.6(8.0,9.1) | 0.621 |
| Minimum calcium(mmol/L) | 7.9(7.4,8.4) | 7.9(7.4,8.4) | 7.9(7.3,8.4) | 0.889 |
| Maximum RBC (m/μL) | 3.9(3.3,4.4) | 3.9(3.3,4.4) | 3.8(3.3,4.4) | 0.071 |
| Minimum RBC (m/μL) | 3.5(3.0,4.0) | 3.5(3.0,4.0) | 3.5(3.0,4.0) | 0.095 |
| Maximum Hemoglobin(g/dL) | 11.3(9.8,13.0) | 11.4(9.9,13.0) | 11.2(9.7,13.0) | 0.219 |
| Minimum Hemoglobin(g/dL) | 10.1(8.6,11.6) | 10.1(8.6,11.7) | 10.0(8.5,11.6) | 0.245 |
| Maximum platelet (×10^3^/μL) | 211.0(147.0,283.0) | 212.0(146.0,284.0) | 209.0(147.0,283.0) | 0.730 |

**Supplementary Table S2. Patients’ baseline characteristics in the post-PSM cohort (n=2478). (Continued)**

| Characteristics | Total | Non-ICU p-LOS, n=1239 | ICU p-LOS,  n=1239 | P |
| --- | --- | --- | --- | --- |
| Minimum platelet (×10^3^/μL) | 177.0(121.0,238.0) | 177.0(120.0,240.0) | 177.0(121.0,234.0) | 0.776 |
| Maximum RDW (%) | 16.0(14.6 ,17.5) | 16.0(14.5,17.5) | 16.0(14.6 ,17.5) | 0.058 |
| Minimum RDW (%) | 16.0(14.4,17.2) | 16.0(14.1,17.1) | 16.0(14.5,17) | 0.117 |
| Maximum white blood cell (×10^3^ /uL) | 18.6(14.3,25.5) | 18.1(12.8,26.1) | 18.9(14.1,25.0) | 0.119 |
| Minimum white blood cell(×10^3^/uL) | 7.4(5.3,10.1) | 7.5(5.6,10.1) | 7.3(5.3,10.0) | 0.590 |
| Maximum MCV (fl) | 91.0(87.0,96.0) | 91.0(87.2,96.0) | 91.2(86.5,96.2) | 0.096 |
| Minimum MCV (fl) | 90.0(86.0,95.0) | 90.0(86.0,94.2) | 90.0(85.6,94.5) | 0.205 |
| Maximum creatinine (µmol/L) | 1.9(1.2,3.1) | 1.8(1.2,3.0) | 1.9(1.2,3.3) | 0.095 |
| Minimum creatinine (µmol/L) | 1.5(1.0,2.4) | 1.4(0.9,2.3) | 1.5(1.0,2.5) | 0.040 |
| Maximum lactate (mmol/L) | 2.5(1.6,3.6) | 2.6(1.6,3.6) | 2.4(1.6,3.6) | 0.056 |
| Minimum lactate (mmol/L) | 1.7(1.4,2.3) | 1.7(1.1,2.4) | 1.7(1.2,2.3) | 0.307 |
| Maximum glucose (mg/dL) | 184.0(138.0,250.0) | 184.0(138.0,254.0) | 181.0(139.0,247.0) | 0.753 |
| Minimum glucose (mg/dL) | 108.0(86.0,134.0) | 108.0(87.0,136.0) | 107.0(86.0,132.0) | 0.117 |
| Maximum BUN (mg/dL) | 35.0(22.0,53.0) | 33.0(22.0,50.0) | 36.0(22.0,55.0) | 0.008 |
| Minimum BUN (mg/dL) | 28.0(18.0,45.0) | 27.0(18.0,42.0) | 30.0(18.0,46.0) | 0.010 |
| Maximum bilirubin (umol/L) | 0.9(0.5,1.5) | 0.9(0.5,1.5) | 0.9(0.5,1.5) | 0.489 |
| Minimum bilirubin (umol/L) | 0.8(0.5,1.3) | 0.8(0.5,1.2) | 0.8(0.5,1.3) | 0.442 |
| Maximum bicarbonate(mmol/L) | 25.0(22.0,28.0) | 25.0(22.0,28.0) | 25.0(22.0,28.0) | 0.429 |
| Minimum bicarbonate(mmol/L) | 22.0(18.0,25.0) | 22.0(18.0,25.0) | 22.0(18.0,25.0) | 0.099 |
| Maximum albumin(mmol/L) | 2.9(2.5,3.4) | 3(2.5,3.5) | 2.9(2.4,3.3) | <0.001 |
| Minimum albumin(mmol/L) | 2,6(2.2,3.0) | 2,6(2.2,3.0) | 2.6(2.1,2.9) | <0.001 |
| Maximum chloride (mmol/L) | 105.0(100.0.109.0) | 105.0(100.0.109.0) | 105.0(100.0.109.0) | 0.452 |
| Minimum chloride (mmol/L) | 100.0(96.0,105.0) | 100.0(96.0,104.0) | 100.0(96.0,105.0) | 0.681 |
| Infection site, n (%) |  |  |  |  |
| Pulmonary | 1206.0(41.4) | 497.0(40.1) | 529.0(42.7) | 0.192 |
| Renal | 535.0(21.6) | 309,0(25.0) | 226.0(18.2) | <0.001 |
| Gastrointestinal tract | 269.0(10.9) | 129.0(10.4) | 140.0(11.3) | 0.477 |
| Skin/soft tissue | 259.0(10.5) | 116.0(9.4) | 143.0(11.5) | 0.076 |
| Others/Unknown | 389.0(15.7) | 188.0(15.1) | 201.0(16.2) | 0.473 |
| Clinical outcome |  |  |  |  |
| ICU LOS, days | 5.0(2.6,8.0) | 2.6(1.8,3.5) | 8.0(6.1,11.5) | <0.001 |
| Hospital LOS, days | 9.2(5.9,14.9) | 6.2(4.1,9.8) | 13.1(8.9,18.7) | <0.001 |
| ICU Mortality, n (%) | 462.0(18.7) | 115.0(9.3) | 149.0(12.0) | 0.027 |
| Hospital Mortality, n (%) | 421.0(16.0) | 182.0(14.7) | 239.0(19.3) | 0.002 |

Notes: Data are expressed as median (IQR), or n (%). Analysis of variance (or the Kruskal-Wallis test) and Chi-square (or Fisher’s exact) tests were used for comparisons among groups. Statistical significance (P<0.05).

**^a^**Severe score is calculated on the first day of each ICU patients’ stay.

^b^Vital signs are calculated on the first 24 h of each ICU patients’ stay.

**^c^**Laboratory results the first result of each patients’ ICU stay.

Abbreviations: PSM, propensity score matching; APACHE IV, acute physiology and chronic health evaluation IV; SOFA, sequential organ failure assessment; GCS, glasgow coma scale; RDW, red cell volume distribution width; RBC, red blood cell; MCV, mean red cell volume; BUN, blood urea nitrogen; ICU, intensive care unit; p-LOS, prolonged length of stay.
