# Supplementary material for: A nomogram to predict prolonged stay of obesity patients with sepsis in ICU: Relevancy for predictive, personalized, preventive, and participatory healthcare strategies
Source: Front Public Health. 2022 Aug 11;10:944790. doi: 10.3389/fpubh.2022.944790 (PMC9403617; doi:10.3389/fpubh.2022.944790)
Supplement: Supplementary file 3 [file Table_3.DOCX]

| Characteristics | Total | Training set  n=3606 | Internal validation set n=1600 | P-value |
| --- | --- | --- | --- | --- |
| Age, years | 65.0(55.0,74.0) | 65.0(55.0,75.0) | 65.0(55.0,74.0) | 0.759 |
| Male, n (%) | 2773.0(53.3) | 1940.0(53.8) | 833.0(52.1) | 0.247 |
| Race, n (%) |  |  |  | 0.533 |
| Caucasian | 4753.0(91.3) | 2878.0(79.8) | 1875.0(79.7) |  |
| American | 605.0(11.6) | 410.0(11.4) | 195.0(12.2) |  |
| Other/Unknown | 448.0(8.6) | 318.0(8.8) | 130.0(8.1) |  |
| BMI, kg/m^2^ | 38.0(32.0,41.0) | 38.0(32.0,41.0) | 38.0(32.0,41.0) | 0.051 |
| LOS before admission to ICU, days | 1.3(0.1,0.4) | 1.3(0.1,0.5) | 1.2(0.1,0.4) | 0.145 |
| Comorbidities, n (%) |  |  |  |  |
| Chronic obstructive pulmonary diseases | 462.0(8,9) | 318.0(8.8) | 144.0(9.0) | 0.832 |
| Chronic kidney disease | 459.0(8.8) | 320.0(8.9) | 139.0(8.7) | 0.827 |
| Liver disease | 286.0(5.5) | 192.0(5.3) | 94.0(5.9) | 0.421 |
| Hypertension | 457.0(8.8) | 331.0(9.2) | 126.0(7.9) | 0.125 |
| Diabetes | 934.0(17.9) | 654.0(18.1) | 280.0(17.5) | 0.581 |
| Congestive heart failure | 504.0(9.7) | 356.0(9.9) | 148.0(9.3) | 0.483 |
| Coronary heart disease | 163.0(3.1) | 108.0(3.0) | 55.0(3.4) | 0.398 |
| Malignant tumors | 207.0(4.0) | 141.0(3.9) | 66.0(4.1) | 0.714 |
| Diabetes | 934.0(17.9) | 654.0(18.1) | 280.0(17.5) | 0.581 |
| Severity score^a^ |  |  |  |  |
| APACHE IV | 66.0(52.0,84.0) | 66.0(51.0,84.0) | 66.0(52.0,83.0) | 0.861 |
| SOFA | 6.0(4.0,9.0) | 6.0(4.0,9.0) | 6.0(4.0,9.0) | 0.698 |
| GCS | 15.0(12.0,15.0) | 15.0(13.0,15.0) | 15.0(12.0,15.0) | 0.670 |
| Vital signs^b^ |  |  |  |  |
| Maximum heart rates (/min) | 113.0(98.0,128.0) | 113.0(99.0.128.0) | 112.0(98.0,128.0) | 0.474 |
| Minimum heart rates (/min) | 76.0(66.0,88.0) | 76.0(66.0,88.0) | 76.0(66.0,87.0) | 0.583 |
| Maximum respiratory rates (/min) | 30.0(26.0,36.0) | 30.0(26.0,36.0) | 30.0(26.0,37.0) | 0.631 |
| Minimum respiratory rates (/min) | 14.0(11.0,16.0) | 14.0(11.0,17.0) | 14.0(11.0,16.0) | 0.202 |
| Maximum mean arterial pressure (mmHg) | 102.0(90.0,117.0) | 102.0(90.0,117.0) | 103.0(91.0,117.0) | 0.326 |
| Minimum mean arterial pressure (mmHg) | 54.0(46.0,63.0) | 54.0(46.0,63.0) | 55.0(46.0,62.0) | 0.526 |
| Maximum temperature (◦C) | 37.5(37.2,38.5) | 37.6(37.1,38.4) | 37.5(37.1,38.5) | 0.155 |
| Minimum temperature (◦C) | 36.2(36.1,36.7 ) | 36.3(36.0,36.7) | 36.2(36.1,36.7) | 0.759 |
| Interventions (1^st^ 24 h), n (%) |  |  |  |  |
| Ventilation, n (%) | 1421.0 (27.3) | 655.0 (18.2) | 766.0(47.9) | 0.652 |
| Vasopressors, n (%) | 2468.0 (47.4) | 1702.0(47.2) | 766.0(47.9) | 0.895 |
| Dialysis, n (%) | 342.0(6.6) | 225.0(6.2) | 117.0(7.3) | 0.149 |
| Laboratory results^c^, (median (IQR)) |  |  |  |  |
| Maximum sodium (mmol/L) | 139.0(136.0,142.0) | 139.0(136.0,142.0) | 139.0(136.0,142.0) | 0.865 |
| Minimum sodium (mmol/L) | 136.0(133.0,139.0) | 136.0(133.0,139.0) | 136.0(133.0,139.0) | 0.889 |
| Maximum potassium (mmol/L) | 4.4(4.0,5.0) | 4.4(4.0,5.0) | 4.4(3.9,4.9) | 0.105 |
| Minimum potassium(mmol/L) | 3.8(3.4,4.3) | 3.9(3.5,4.3) | 3.8(3.4,4.3) | 0.557 |
| Maximum calcium (mmol/L) | 8.6(8.0,9.1) | 8.6(8.0,9.1) | 8.5(8.0,9.1) | 0.303 |
| Minimum calcium (mmol/L) | 7.9(7.4,8.4) | 7.9(7.4,8.4) | 7.9(7.4,8.4) | 0.288 |
| Maximum RBC(m/μL) | 3.9(3.3,4.4) | 3.9(3.3,4.4) | 3.9(3.3,4.4) | 0.780 |
| Minimum RBC (m/μL) | 3.5(3.0,4.0) | 3.5(3.0,4.0) | 3.5(3.0,4.0) | 0.746 |
| Maximum Hemoglobin(g/dL) | 11.4(9.8,13.1) | 11.4(9.7,13.0) | 11.5(9.9.13.2) | 0.119 |
| Minimum Hemoglobin(g/dL) | 10.1(8.7,11.7) | 10.1(8.6,11.7) | 10.2(8.7,11.8) | 0.118 |
| Maximum platelet (×10^3^/μL) | 222.0(148.0,281.0) | 223.0(148.0,282.0) | 222.0(148.0,279.0) | 0.660 |
| Minimum platelet(×10^3^/μL) | 187.0(122.0,238.0) | 188.0(123.0,239.0) | 186.0(118.0,235.0) | 0.429 |
| Maximum RDW (%) | 15.8(14.3,17.4) | 15.8(14.3,17) | 15.9(14.2 ,17.5) | 0.358 |
| Minimum RDW (%) | 15.5(14.3,17) | 15.6(14.2,16.9) | 15.5(14.5,17) | 0.364 |
| Maximum white blood cell (×10^3^ /uL) | 17.3(12.5,24.1) | 17.3(12.5,24.1) | 17.5(12.6,24.0) | 0.486 |
| Minimum white blood cell(×10^3^/uL) | 7.9(5.6,10.5) | 7.7(5.6,110.7) | 7.9(5.6,10.7) | 0.309 |
| Maximum MCV (fl) | 91.0(87.0,96.0) | 91.0(87.0,96.0) | 91.0(87.0,95.0) | 0.060 |
| Minimum MCV (fl) | 90.0(85.0,94.0) | 90.0(86.0,94.0) | 90.0(86.0,94.0) | 0.064 |
| Maximum creatinine (µmol/L) | 1.8(1.15,3.0) | 1.8(1.2,3.1) | 1.8(1.14,3) | 0.205 |
| Minimum creatinine (µmol/L) | 1.4(0.9,2.4) | 1.4()0.9,2.4 | 1.4(0.9,2.3) | 0.311 |
| Maximum lactate (mmol/L) | 2.5(1.6,3.7) | 2.5(1.6,3.7) | 2.5(1.6,3.7) | 0.435 |
| Minimum lactate (mmol/L) | 1.7(1.1,2.3) | 1.7(1.1,2.3) | 1.6(1.1,2.3) | 0.439 |
| Maximum glucose (mg/dL) | 180.0(137.0,247.0) | 180.0(137.0,247.0) | 180.0(138.0,251.0) | 0.822 |

**Supplementary Table S3. Baseline characteristics of patients in training set and validation set (n = 5206).**

**Supplementary Table S3. Baseline characteristics of patients in training set and validation set (n = 5206). (Continued)**

| Characteristics | Total | Training set  n=3606 | Internal validation set n=1600 | P-value |
| --- | --- | --- | --- | --- |
| Minimum glucose (mg/dL) | 106.0(86.0,133.0) | 106.0(87.0,134.0) | 108.0(86.0,132.0) | 0.112 |
| Maximum BUN (mg/dL) | 34.0(21.0,52.0) | 34.0(22.0,52.0) | 34.0(21.0,52.0) | 0.405 |
| Minimum BUN (mg/dL) | 33.0(17.0,43.0) | 33.0(18.0,43.0) | 32.0(17.0,43.0) | 0.562 |
| Maximum bilirubin (umol/L) | 0.9(0.5,1.5) | 0.9(0.5,1.6) | 0.9(0.5,1.5) | 0.200 |
| Minimum bilirubin (umol/L) | 0.8(0.5,1.3) | 0.8(0.5,1.3) | 0.8(0.4,1.3) | 0.450 |
| Maximum bicarbonate (mmol/L) | 24.3(21.0,27.0) | 25.0(22.0,28.0) | 24.7(21.0,27.0) | 0.242 |
| Minimum bicarbonate (mmol/L) | 22.-0(18.0,25.0) | 22.0(18.0,25.0) | 22.0(18.0,25.0) | 0.971 |
| Maximum albumin(mmol/L) | 3.0(2.5,3.4) | 3.0(2.5,3.5) | 3.0(2.5,3.4) | 1.000 |
| Minimum albumin(mmol/L) | 2,6(2.2,3.0) | 2.6(2.2,3.0) | 2,6(2.2,3.0) | 0.504 |
| Maximum chloride (mmol/L) | 105.0(101.0,109.0) | 104.0(100.0.109.0) | 105.0(101.0,109.0) | 0.352 |
| Minimum chloride (mmol/L) | 100.0(96.0,105.0) | 100.5(96.0,105.0) | 100.0(96.0,105.0) | 0.905 |
| Clinical outcome |  |  |  |  |
| ICU LOS, days | 3.0(1.9,5.6) | 3.0(1.9,5.6) | 3.0(1.9,5.6) | 0.649 |
| Hospital LOS, days | 7.3(4.6,12.2) | 7.3(4.6,12.3) | 7.3(4.6,12.2) | 0.637 |
| ICU Mortality, n (%) | 456.0(8.8) | 298.0(8.3) | 158.0(9.9) | 0.058 |
| Hospital Mortality, n(%) | 726.0(13.9) | 497.0(13.8) | 229.0(14.3) | 0.611 |

Notes: Data are expressed as median (IQR), or n (%). Analysis of variance (or the Kruskal-Wallis test) and Chi-square (or Fisher’s exact) tests were used for comparisons among groups. Statistical significance (P<0.05).

**^a^**Severe score is calculated on the first day of each ICU patients’ stay.

^b^Vital signs are calculated on the first 24 h of each ICU patients’ stay.

**^c^**Laboratory results the first result of each patients’ ICU stay.

Abbreviations: BMI, body mass index; APACHE IV, acute physiology and chronic health evaluation IV; SOFA, sequential organ failure assessment; GCS, glasgow coma scale; ICU, intensive care unit; LOS, length of stay.
