# Supplementary figures and images for: A nomogram to predict prolonged stay of obesity patients with sepsis in ICU: Relevancy for predictive, personalized, preventive, and participatory healthcare strategies
Source: Front Public Health. 2022 Aug 11;10:944790. doi: 10.3389/fpubh.2022.944790 (PMC9403617; doi:10.3389/fpubh.2022.944790)

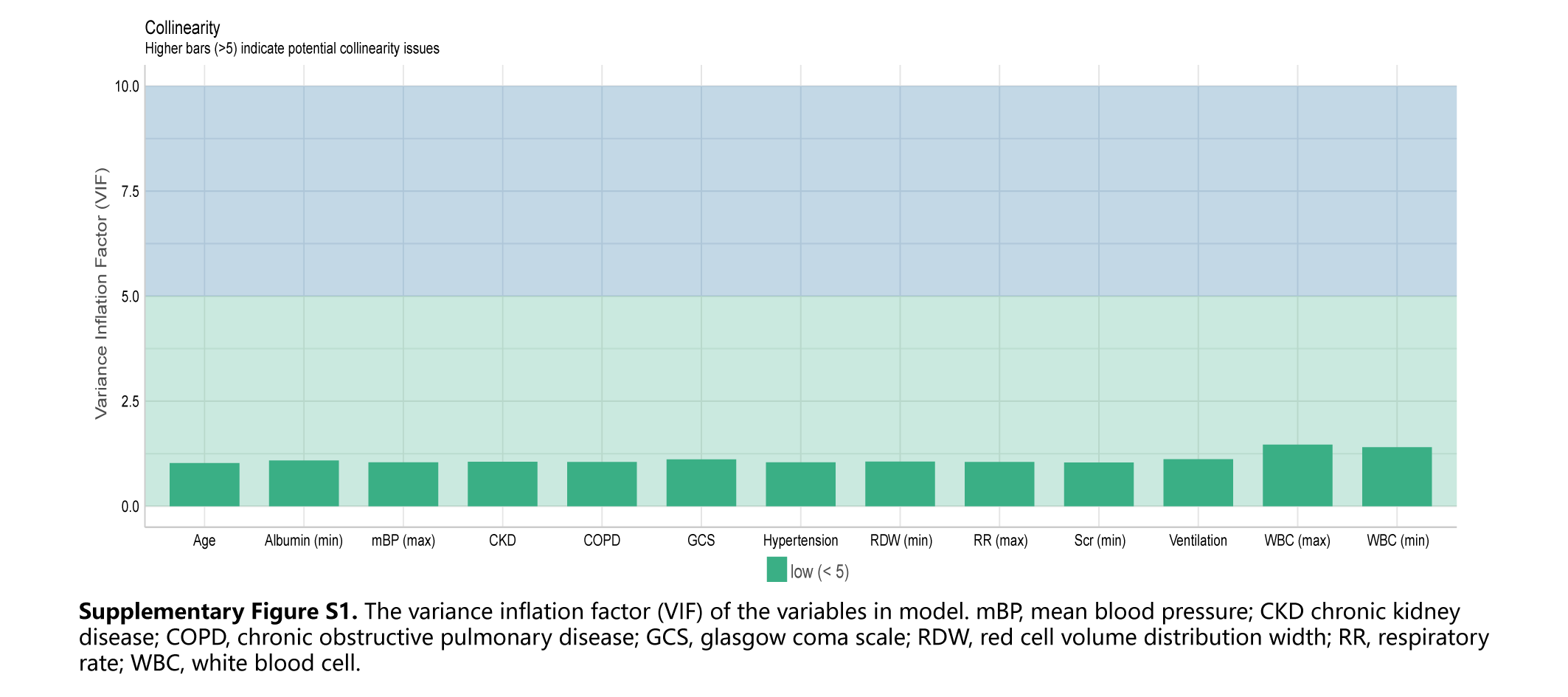

Supplement: Supplementary file 4 [file Image_1.TIF]

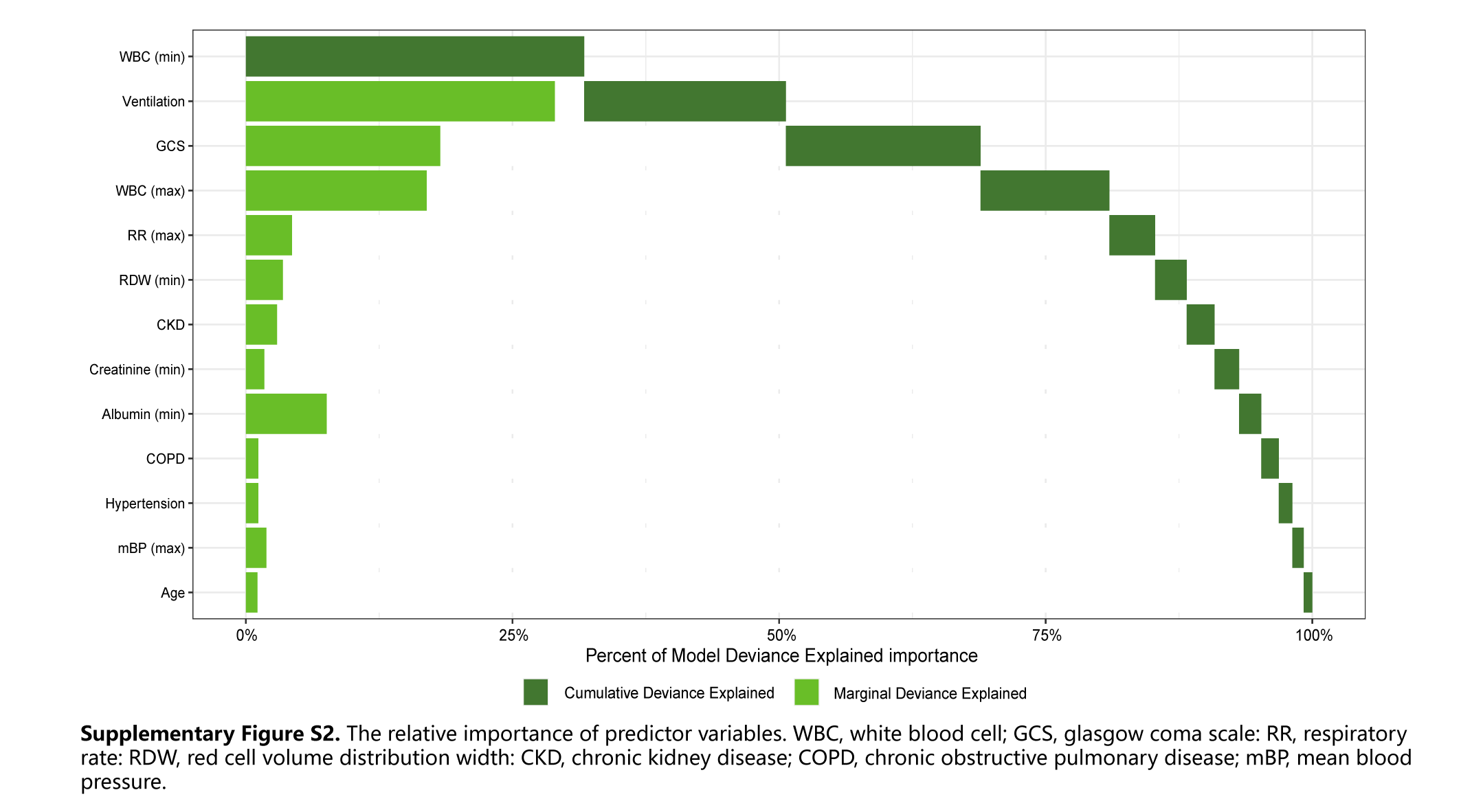

Supplement: Supplementary file 5 [file Image_2.TIF]
